# Elucidation of user autonomous driving system preference mechanisms under the extension of internal and external factors

**DOI:** 10.1038/s41598-025-15442-6

**Published:** 2025-08-17

**Authors:** Juncheng Mu, Linglin Zhou

**Affiliations:** School of Fine Arts, Nanjing Normal University, Nanjing, 210023 China

**Keywords:** Advanced driver assistance systems (ADAS), Driverless systems, Sustained usage intention, Comparative study, Behavior intention, Human behaviour, Computer science

## Abstract

This study is based on the extended Technology Acceptance Model, integrating Habit Theory and Regret Theory to construct a model of users’ continuous usage intentions. It conducts a comparative analysis of users’ continuous usage intentions for both assisted driving systems and driverless systems. Data was collected through an online questionnaire survey and analyzed using Structural Equation Modeling. The results indicate that, within assisted driving systems, perceived importance and driving habits significantly influence continuous usage intentions; conversely, in driverless systems, driving habits are paramount. In both systems, users’ perceived importance and experience of regret have a significant impact on driving habits, with experience of regret indirectly affecting continuous usage intentions through driving habits. User scale exerts direct or indirect effects through various variables. Regarding control variables, significant differences exist between the two systems; users of assisted driving systems prioritize economic benefits, while users of driverless systems focus on after-sales service. This research theoretically establishes a new framework, enriching and refining relevant theories; practically, it provides references for system improvement and promotion; and it suggests avenues for future research into control variables and segmented user groups. The study reveals the driving factors behind users’ continuous usage of different driving systems, constructing a universally applicable theoretical model with significant academic and practical implications.

## Introduction

In recent years, urban transportation has experienced rapid development, yet traffic congestion has become increasingly severe. Research estimates that congestion leads to economic losses of up to nearly 100 billion euros annually, according to European Union statistics^1^. In China, economic losses directly attributable to traffic congestion account for 5–8% of the GDP, reaching 250 billion yuan (approximately 40 billion USD at the time)^2^. Congestion also increases energy consumption and tailpipe emissions. Fuel consumption of gasoline vehicles on urban roads increases by 20–200% under congested conditions^3^, leading to a corresponding increase in carbon emissions. Pollutant emissions during peak congestion can be up to 10 times that of normal conditions^4^, posing a significant challenge to residents’ quality of life and the sustainable development of cities. Predictions indicate that by 2050, urban transportation carbon emissions can be reduced by 34% through disruptive reforms such as autonomous driving^5^, vehicle electrification, and shared transportation. Autonomous driving optimizes driving behavior and route planning, improving fuel efficiency and reducing per-mile emissions, making its importance in addressing urban traffic problems self-evident^6^. Against this backdrop, the prototype of early autonomous driving systems—Advanced Driver-Assistance Systems (ADAS)—emerged. Autonomous driving technology has entered a critical development window, achieving significant progress and breakthroughs, particularly in cutting-edge fields such as artificial intelligence, big data analysis, and the Internet of Things (IoT)^7^.The early prototype of autonomous driving systems—the Advanced Driver Assistance Systems (ADAS)—has emerged in response to this need. As core components of modern automotive technology, both ADAS and autonomous driving systems aim to enhance road safety and driving comfort. ADAS integrates a variety of safety and convenience features, providing comprehensive support to drivers, effectively reducing or even eliminating operational errors, thereby improving traffic and transportation efficiency^8–10^. ADAS includes a range of practical functions such as Adaptive Cruise Control (ACC), Automatic Emergency Braking (AEB), Blind Spot Detection (BSD), Lane Departure Warning (LDW), and Traffic Sign Recognition (TSR)^11–13^. Research indicates that the ADAS within autonomous driving systems can significantly alleviate driver workload, enhance driving safety, and reduce the incidence of traffic accidents^8,9^. Autonomous driving systems take this a step further by utilizing advanced technologies such as environmental perception, automatic control, and path navigation to achieve vehicle autonomy. The technological advancements in this area are attributed to significant breakthroughs in artificial intelligence, big data analytics, machine learning, and computer vision^7,14^. Furthermore, autonomous driving systems not only help alleviate traffic congestion but also improve fuel efficiency, thereby further ensuring driving safety^7^.

While both ADAS and driverless systems fall under the umbrella of autonomous driving, there are distinctions between the two. Current research has produced a wealth of findings regarding ADAS within the realm of automated driving systems. Features such as lane departure warnings, forward collision warnings, and blind spot detection in ADAS can significantly reduce the incidence of fatal and severe accidents^15^. Studies indicate that vehicles equipped with these ADAS features have a lower probability of experiencing frontal collisions and rear-end accidents compared to those without such technologies^15^. Research conducted in the United States on BMW models found that vehicles equipped with automatic emergency braking and lane departure warning systems are 23% less likely to be involved in moderate or severe accidents than those lacking these technologies, thereby demonstrating the effectiveness of ADAS technology in real-world driving environments^16^. Furthermore, a study based on data from German insurers estimated that Collision Mitigation Braking Systems (CMBS) and lateral guidance systems (including lane change assistance and lane keeping assistance) can prevent up to 17.8% and 7.3% of accidents, respectively, highlighting the substantial potential of ADAS in assisting drivers to reduce accidents^17^. In contrast, driverless systems achieve complete automation of vehicle control, with the driver functioning more as a passenger in such scenarios. From a technological standpoint, achieving driverless necessitates highly advanced computer vision, deep learning, and artificial intelligence technologies^18^. For instance, Tesla’s Full Self-Driving (FSD) computer is based on a new system-on-chip (SoC) that integrates a CPU, ISP, GPU, and custom neural network accelerators, capable of processing data at rates of up to 2300 frames per second, which is 21 times that of its predecessor^19^. In terms of safety and efficiency in vehicle usage, driverless systems significantly contribute to reducing traffic accidents and enhancing road utilization efficiency^19,20^. As illustrated in Table 1, significant distinctions exist between ADAS and Driverless Systems across various dimensions, including functionality, technical specifications, safety protocols, and user experience.

**Table 1:** Comparative Analysis of Assisted Driving Systems and Driverless Systems.

Dimensions	Advanced driver assistance systems (ADAS)	Driverless systems
Technological dependency	Relying solely on onboard sensors such as radar and cameras, the system can directly achieve functionalities without external communication^21^	V2X communication, dependence on high-precision maps, and redundant systems enabled by AI algorithms facilitate real-time decision-making^22^
Security	ADAS reduces driver distraction risks by monitoring vehicle status and environment in real-time, effectively decreasing collision likelihood, though it cannot fully replace human judgment^23^	Theoretically, it can eliminate human driving errors such as fatigue and distraction, significantly reducing traffic accidents, but must address extreme scenarios like sudden obstacles^22^
Cost	Cost-effective and easy to integrate, these systems are widely adopted in the market^24^	Hardware components such as LiDAR and multi-sensor fusion systems, along with software like AI decision-making algorithms, entail high costs, necessitating redundant design and ongoing research and development^25^
Application scenarios	Suitable for various traffic scenarios, including highways and low-speed environments^26^	It can cover all scenarios but requires infrastructure improvements^27^
Features of use	Drivers need to adapt to system limitations; ADAS may diminish driving enjoyment, and user trust in these systems varies^23^	Long-term education and pilot programs are essential, relying on comprehensive infrastructure such as V2I communication and high-precision maps. Currently in the pilot stage, public acceptance remains relatively low^22^

From a functional and technical perspective, ADAS primarily target limited application scenarios, with core functionalities aimed at assisting drivers in better vehicle control. Common features include Adaptive Cruise Control (ACC), Lane Departure Warning (LDW), and Automatic Emergency Braking (AEB)^21,28,29^. These systems utilize onboard sensors and cameras to perceive the surrounding environment, effectively alleviating the driver’s workload by issuing warnings or executing automated control tasks^11,21,28^. In contrast, driverless systems operate without driver intervention and can handle a broader and more complex range of driving scenarios^30^. Such systems not only rely on highly advanced sensors and algorithms but also require communication capabilities between the vehicle and the external environment, typically achieved through existing Vehicle-to-Everything (V2X) technology^29,31^.

In terms of safety performance, ADAS significantly enhances road safety by reducing the likelihood of human error^32^. However, its safety effectiveness may vary based on usage conditions and driver behavior patterns during actual operation^33^. Driverless systems further strengthen safety through the integration of more advanced sensors and algorithms, along with real-time communication capabilities with the external environment^7,31^. Consequently, the development and testing of driverless systems entail more stringent and complex requirements compared to ADAS. This is because driverless systems must ensure safe and stable operation under a variety of complex and extreme conditions, rather than being limited to specific driving scenarios^29,31^. This necessitates extensive simulation testing and real-world road testing to ensure the reliability and safety of the system^29,31^.

In terms of safety performance, ADAS systems significantly enhance road safety by reducing the probability of human error^32^. A critical factor for autonomous driving systems is the ability to make reliable real-time decisions, making technological reliability a key aspect of these systems^34^. This technological reliability not only improves vehicle energy efficiency but also provides a safer and more comfortable driving experience^35^. Furthermore, advancements in artificial intelligence and deep learning have enabled driving systems to demonstrate improved safety in pedestrian detection, obstacle recognition, and stereo matching^36,37^. However, the actual safety performance can vary depending on usage conditions and driver behavior^33^. Full autonomous driving systems further enhance safety through advanced sensor and algorithm integration, along with real-time communication capabilities with the external environment^7,31^. Consequently, the development and testing requirements for full autonomous driving systems are more stringent and complex compared to ADAS. This is because full autonomous driving systems must ensure safe and stable operation in various complex and extreme conditions, not just specific driving scenarios^29,31^. This necessitates extensive simulation and real-world road testing to ensure system reliability and safety^29,31^.

From the perspective of user experience, there is a fundamental distinction between the two. ADAS are typically regarded as tools that assist in driving, with the driver remaining the primary operator of the vehicle^38^. As these assistance systems continue to evolve, the driver’s reliance on them is likely to increase, which may impact their driving experience and trust in the system^39,40^. In contrast, driverless systems completely replace the driver’s role, transforming them into a passenger, which inevitably raises concerns among users regarding safety issues^41^. Despite the rapid advancements in both types of autonomous driving systems in recent years, they face numerous challenges and controversies, particularly concerning safety, ethical considerations, social acceptance, and technological security^42^.

Current research on users’ continued intention to use autonomous driving systems remains in the exploratory stage, although studies on system acceptance are relatively more abundant. Findings indicate that perceived usefulness and perceived ease of use are positively correlated with users’ acceptance of autonomous driving systems^43^. Perceived usefulness refers to the extent to which users believe the technology can meet their needs^44^. In the context of autonomous vehicle applications, users are more inclined to accept and adopt the system when they perceive it can enhance driving advantages^45^. Perceived ease of use involves users’ evaluations of the simplicity and intuitiveness of the technology’s operation^44,46^. When users find the autonomous system easy to understand and operate, their acceptance and willingness to continue using it tend to increase^45^. Additionally, research employing the Technology Acceptance Model (TAM) and other related frameworks has confirmed that users’ acceptance of new autonomous driving technologies is significantly influenced by these perceptions^47,48^. Other theoretical models have also been applied in the automotive domain, such as the Transtheoretical Model (TTM), which explores how attitude factors affect individuals’ perceptions of shared mobility services^49^, and studies on the impact of flexible-route public transportation systems on psychological factors^50^. Furthermore, the Partially Proportional Odds (PPO) model has been used to investigate factors influencing motor vehicle accidents^51^. These studies collectively demonstrate that the effective and rational application of new technologies can not only improve user efficiency in adopting these innovations but also significantly reduce accident rates. Users’ perceptions of the usefulness of new technologies, along with their cognition and attitudes toward these systems, play a crucial role in determining acceptance. User experience is also a key factor influencing willingness to use autonomous driving systems; employing VR virtual driving technology can enhance user acceptance and foster greater trust in autonomous systems^52^. Based on existing theoretical research, it is evident that users’ perceptions and experiences are critical determinants of their acceptance and continued use of autonomous driving systems. Therefore, exploring the impact of different types of autonomous driving systems on users’ sustained usage intentions is of significant importance, offering practical value and guidance for future development and technological improvements. Currently, research on user continued usage behavior across various autonomous driving system types is still in its infancy, and the technology itself remains in the refinement and initial implementation stages. It is particularly necessary to delve deeper into the influencing factors and mechanisms underlying users’ continued use intentions for different autonomous system types, develop comparative models, and tailor improvements based on user needs for each system. Additionally, exploring future directions for autonomous driving technology remains a vital area of research.

This study aims to fill the research gap in the relevant field by thoroughly examining the impact of ADAS and Driverless driving systems on users’ continuous usage intentions. Specifically, it seeks to: first, clarify the differences between the two system types in key factors; second, identify the core determinants influencing users’ sustained engagement with both systems; third, develop a comparative model of users’ continuous usage intentions; and fourth, propose improvement directions for both systems based on user needs, thereby providing a solid theoretical foundation and practical guidance for the long-term development and market promotion of autonomous driving technology. This research will contribute to the ongoing refinement and innovation of autonomous driving systems, enhance users’ willingness to continue using these systems, and promote their widespread adoption and application. Grounded in the Technology Acceptance Model (TAM), the study constructs a three-tier analytical framework comprising “control variables (system factors), technology acceptance (user perceptions), and continuous usage behavior.” It focuses on core user perceptions such as perceived usefulness, perceived ease of use, and safety. From six dimensions—economic, technological, safety, ethical, regulatory, and social—it conducts an in-depth comparison of ADAS and driverless driving systems to deconstruct their differences. Through empirical research, the study investigates the influencing factors and mechanisms affecting users’ continuous usage intentions for different autonomous driving systems, revealing the intrinsic relationship between the two system types and user retention. A scientifically rigorous survey questionnaire will be designed to collect data on user experiences, perceptions, and usage intentions. Statistical methods such as structural equation modeling will be employed to rigorously test theoretical hypotheses, explore key factors and pathways influencing continuous usage intentions, and ensure the scientific validity and reliability of the findings. Furthermore, this research transcends the limitations of existing studies that predominantly focus on single autonomous driving systems or user acceptance, adopting a comparative perspective to explore the impact of both system types on user retention. It fills a significant research gap in the field and opens new avenues for understanding user behavior in autonomous driving technology.

Given the significance of this, it is crucial to investigate the impact of two distinct autonomous driving systems on users’ continuous usage intentions. This research holds practical value and offers a reference point for guiding the future development and technological enhancements of autonomous driving systems. It is essential to delve into the factors and mechanisms influencing users’ continuous usage intentions for different types of autonomous driving systems and to construct a new comparative research model. This will facilitate the improvement and refinement of both systems based on user needs, while also exploring the future development directions of autonomous driving systems. This study will contribute to the continuous improvement and innovation of autonomous driving systems, thereby enhancing users’ willingness to use them continuously and promoting their widespread adoption. The overall structure of this research is as follows: Section “Literature review” will systematically review and elaborate on the control variables and relevant theoretical literature of autonomous driving systems; Section “Research hypotheses” will focus on proposing research hypotheses and constructing a theoretical model; Section “Research framework, approaches and questionnaire design” will detail the research organization, implementation process, and questionnaire design; Sections “Empirical study 1: research on the continuous use intention of the users of the driver assistance system” and “Empirical study 2: continuous user intent research for driverless system” will present the analysis of empirical data; Section “Discussion” will discuss the research findings; Section “Theoretical and implications” will elaborate on the theoretical significance and practical contributions of the research; and finally, Section “Conclusions” will summarize the research and draw conclusions.

## Literature review

### Control variable of automatic driving system

The extant literature indicates that technological reliability manifests in the domains of automotive energy management, battery utilization, and intelligent driving algorithms, thereby enhancing vehicle operational efficiency and economic viability^53^. This encompasses the performance of autonomous driving systems, including their dependability (e.g., frequency of malfunctions) and compatibility with other traffic systems^54^. The quality of this technology directly influences user adoption of the system. Furthermore, economic benefits constitute a critical determinant of user engagement with autonomous driving systems. The costs associated with acquiring vehicles equipped with autonomous driving systems, maintenance expenses, insurance premiums, and potential economic savings (e.g., reduced fuel consumption, enhanced travel efficiency leading to time savings)^55^ all contribute to users’ willingness to utilize these systems. The implementation of these systems can effectively reduce traffic accidents, increase road capacity, and improve overall efficiency, leading to substantial economic advantages^56^. Moreover, autonomous driving systems can efficiently avoid traffic congestion by identifying better routes, saving both time and fuel, which results in significant economic benefits^57^. Furthermore, the safety of autonomous driving systems is a major concern among users^42^. Enhancing safety is a crucial factor, as there have been ongoing questions regarding safety issues associated with the use of autonomous driving systems. The integration of advanced driver-assistance systems can effectively reduce human error and improve driving safety^10,58^. Safety is a significant challenge for autonomous driving systems, with issues related to safety consistently arising in practical applications^59^. Users’ primary concern revolves around the safety assurances provided by autonomous driving systems during operation; higher safety levels correlate with increased likelihood of sustained system utilization^60^. Social influences also play a significant role, with societal norms and the behaviors of others significantly impacting individual acceptance^61^. Widespread societal endorsement of autonomous driving technology, coupled with supportive legal and regulatory frameworks, further enhances user acceptance and continued usage^62^. In essence, the adoption of autonomous driving systems by peers and positive societal evaluations contribute to increased user acceptance. Post-sale and technical support services are also critical determinants of continued use, encompassing software system management, real-time data processing, and the complexities of experimental and road testing^63–65^. These after-sales services are crucial for consumers’ daily usage. High-quality after-sales service fosters user confidence, ensuring prompt issue resolution and thereby increasing the probability of continued autonomous driving system use^66^. Ethical considerations also significantly influence users’ decisions, including the investigation of accident causes and preventative strategies^67^. For instance, the ability of autonomous driving systems to make correct decisions in emergencies, adhering to human ethical standards, is paramount^68^. Ethical dilemmas, such as decision-making ethics in unavoidable collision scenarios (e.g., prioritizing the safety of vehicle occupants versus pedestrians), data privacy (safeguarding user driving data from misuse), and responsibility attribution (determining liability in accidents among vehicle manufacturers, software developers, and users), all impact user intentions^69^.

Based on these findings, economic, technological, security, ethical, social, and after-sales service factors were incorporated as control variables in the relevant research and analysis. These factors can be directly utilized to predict users’ continued use of autonomous driving systems. In other words, by considering and analyzing these factors, it is possible to determine users’ future intentions to continue using autonomous driving systems, as they are directly correlated with users’ intentions for sustained usage.

### Theoretical model

This research is grounded in the Technology Acceptance Model (TAM), which serves as its foundational framework. The TAM seeks to elucidate and forecast users’ acceptance of information technology, primarily emphasizing two key variables: perceived usefulness and perceived ease of use. These variables significantly impact users’ decisions to adopt and engage with information technology^44^. However, as research has progressed, it has become evident that these two variables alone do not comprehensively explain all instances of technology acceptance. As a result, scholars have begun to investigate additional factors that may influence technology acceptance, integrating elements such as social influence, cognitive processing, and perceived user resources into an extended TAM model^70–72^. Building on this theoretical groundwork, the current study incorporates habit theory and regret theory, examining user scale, perceived importance, and post-experience regret as antecedent variables. These antecedent variables are posited to affect the intention for continued use through user motivation and usage habits. Furthermore, the model considers the potential effects of external factors, including economic, security, technological, ethical, and social influences, as well as service-related aspects, in developing a model of sustained usage behavior for autonomous driving systems. This approach aims to enhance our understanding of the factors and pathways that shape users’ intentions for ongoing engagement with autonomous driving systems.

User habits have received limited attention in the literature concerning specific systems, yet they are extensively examined in disciplines such as social psychology, marketing, consumer behavior, and organizational behavior^73^. In the context of autonomous driving systems, tailoring driving experiences is essential for improving user safety and comfort^74,75^. For example, understanding users’ preferred driving styles and adapting autonomous driving behaviors and vehicle control modes accordingly can significantly enhance passenger satisfaction^76^. Moreover, leveraging artificial intelligence to enable smart vehicles to learn user habits and preferences represents a vital avenue for enhancing user experience^77^. Concurrently, the Technology Acceptance Model (TAM) posits that user acceptance of information technology is influenced by perceived usefulness and perceived ease of use^44^. In light of driving habits, if users perceive that a specific driving system can effectively improve safety and convenience, they are more inclined to continue utilizing these systems^78^. Thus, the development of habits emerges as a crucial factor affecting the intention to persist in usage. Once established, habits can be difficult to alter, even when users recognize their potential adverse effects^79,80^. As a result, ongoing usage behavior is shaped by the interaction of rational cognition and habitual tendencies^81^. On one hand, rational users engage with autonomous driving systems to better achieve their objectives, and they are likely to maintain their usage only when they perceive these systems as beneficial or significant^82^. On the other hand, researchers contend that conscious usage is also influenced by irrational factors, including mood, satisfaction, and other emotional variables^81^. Consequently, positive user experiences promote intentions for continued usage, while negative emotions following the experience, such as regret, may impede such intentions^83^. Traditional behavioral theories consistently assert that cognitive and emotional responses play a role in the formation of continued intentions. However, users frequently engage in habitual behaviors unconsciously while interacting with certain systems^73,81^. Therefore, within the Technology Acceptance Model (TAM), habits are recognized as a pivotal factor mediating satisfaction, usability, significance, convenience, and continued use, underscoring the critical role of habits in user retention^80^.

Furthermore, a substantial body of literature underscores the significant influence of control variables on user acceptance and usage behavior concerning autonomous driving systems, encompassing dimensions such as safety, social impact, service quality, and social trust ^47,84^. Research indicates that social impact is a pivotal factor in predicting the widespread adoption of autonomous vehicles, exerting a more pronounced effect than other variables ^85^. Regarding social trust, numerous studies confirm its direct impact on users’ fundamental acceptance of autonomous driving technology, as well as its indirect effects through a “perceived benefits—risk assessment” dual pathway: enhancing users’ recognition of technological value while mitigating potential risk concerns^86^. Consequently, trust mechanisms are widely acknowledged as critical decision variables in users’ acceptance of autonomous vehicles^87^. Trust directly influences users’ expectations of technological reliability and reinforces acceptance through a risk-buffering mechanism^86^. In consumer contexts, trust serves as a core driver for the market penetration of autonomous vehicles, operating through both direct and indirect effects on attitude transmission^88^. Given the significant impact of external control variables on user acceptance behavior, their potential influence on users’ sustained usage intentions for autonomous driving systems is also evident. This suggests that a systematic examination of the dynamic mechanisms of these control variables is essential when exploring users’ long-term usage behavior.

Moreover, the pertinent literature suggests that external variables substantially affect the acceptance and utilization behaviors associated with autonomous driving systems. These variables encompass safety considerations, social influence, service quality, and social trust^47,84^. For example, social influence is identified as the most significant predictor of public acceptance of autonomous vehicles^85^. Furthermore, social trust exerts both direct and indirect effects on the acceptance of autonomous technology, influencing not only the overall acceptance of such systems but also shaping acceptance through perceived benefits and risks^86^. Additionally, trust is recognized as a critical factor in user acceptance of autonomous vehicles^87^. It directly impacts expectations regarding the technological reliability of these systems and is essential for mitigating perceived risks, thereby directly affecting user acceptance^86^. Trust also plays a crucial role in consumer adoption of autonomous vehicles, influencing adoption through both direct and indirect effects on attitudes^88^. Control Variables significantly impact user acceptance of autonomous driving systems, and as a result, these factors will also affect users’ intentions for continued engagement with automotive autonomous driving systems.

Based on this, this paper extends the Technology Acceptance Model (TAM) by introducing “habit” as a key mediating variable and integrating both rational factors (perceived importance) and irrational factors (post-experience regret) in influencing continuous usage. Traditional TAM primarily explains user acceptance behavior through perceived usefulness and perceived ease of use, but this study emphasizes the critical role of “habit” within the TAM framework, viewing it as an intermediary linking various variables to sustained usage behavior. Furthermore, the model refines the driving mechanisms behind continuous use by incorporating “perceived importance” at the rational level, highlighting that users continue to use autonomous driving systems due to their usefulness in achieving goals (aligning with TAM’s perceived usefulness and ease of use). At the irrational level, emotional variables such as positive experience and post-experience regret are included, indicating that emotions can influence habits and behavioral intentions. This extension breaks through the traditional TAM’s sole focus on rational cognition, integrating irrational factors to enrich the explanation of user continuous usage behavior. It makes TAM more applicable to the context of autonomous driving systems, especially enhancing its explanatory power regarding long-term “continued use,” which is beyond the scope of the conventional TAM model limited to initial acceptance. Therefore, this study adopts a comprehensive approach combining habit theory and regret theory, using user scale, perceived importance, and post-experience regret as antecedent variables. It examines how users’ driving and usage habits influence their continued intention, while incorporating economic, safety, technological, ethical, social influence, and service factors as control variables within the model. This constructs a comprehensive model of continuous usage behavior for autonomous driving systems, aiming to more accurately analyze the key factors and pathways affecting users’ sustained usage intentions, thereby providing valuable insights for research and practice in the autonomous driving field.

## Research hypotheses

### The user scale of the autonomous driving system

The expansion of the user base significantly affects the functionality and user-friendliness of the system. As the number of users interacting with the same platform increases, various factors may be altered due to resource allocation, system load, or other external variables, thereby influencing users’ perceptions and experiences^89^. The significance of user experience in systems and services is continually on the rise^90^. With a growing user population, the system is required to handle a larger volume of data, which can lead to a decline in performance, subsequently impacting user experience. Users’ evaluations of the system transition from initial curiosity to assessments of practicality and usability^91^. According to social cognitive theory, the size of the user group can affect individuals’ self-efficacy and collective efficacy, which in turn influences their intent to engage. This indicates that as the user count increases, individuals perceive the strength of the collective, enhancing their willingness to utilize the system^92^. Therefore, an expanding user base can elevate perceived significance by enhancing both individual and collective efficacy^92^. Additionally, user engagement is vital in increasing perceived importance; active participation in the system can lead to improved utilization rates and overall satisfaction^93^. The ongoing growth of the user base complicates system management, emerging as a critical factor in shaping perceived significance^94^. Therefore, the larger the user scale of different driving systems, the greater the perceived importance of such autonomous driving systems.

#### **H1**

*The user size of automotive assisted driving system has a positive impact on the perceived importance of users.*

#### **H2**

*The user size of a driverless system has a positive impact on the perceived importance of the user.*

Expectation Confirmation Theory posits that user satisfaction and the intention to continue utilizing a system are significantly influenced by the alignment between pre-use expectations and post-use experiences^82^. Specifically, as the user base expands and the system adeptly accommodates a greater number of users while preserving high performance and service quality, user satisfaction tends to rise, fostering positive emotional experiences. In contrast, any failure to uphold these standards can lead to diminished satisfaction and adverse emotional responses, ultimately impacting users’ willingness to persist with the system^82,95^. Following interactions with autonomous driving systems, users may experience either positive or negative emotions, resulting in emotional benefits or feelings of regret, which in turn shape the overall consumer experience. Consequently, this article posits that an increasing number of users engaging with autonomous driving systems fosters positive interactions regarding these experiences, thereby decreasing the likelihood of regret among other users.

#### **H3**

*There is a negative correlation between the user scale of automotive assisted driving system and user experience regret.*

#### **H4**

*There is a negative correlation between user size and user experience regret in driverless systems.*

The preceding statements indicate that the magnitude of a system’s user base is positively correlated with the perceived significance and satisfaction derived from the system^92–94^. Furthermore, the importance and satisfaction of users are critical determinants that shape their habits^82,89,90,95^. Consequently, there exists a positive correlation between the user base of autonomous driving systems and the driving behaviors of users. For a considerable number of drivers, a robust user base enhances communication and interaction during periods when they are not driving, which in turn improves users’ perceptions of the significance and satisfaction associated with autonomous driving systems, ultimately fostering the development of their behavioral patterns.

#### **H5**

*There is a positive correlation between the user scale of automotive assisted driving system and user habits.*

#### **H6**

*There is a positive correlation between user size and user habits of driverless systems.*

### User perceived importance

Davis posits that users’ perceptions regarding the utility and user-friendliness of information technology are pivotal factors influencing their acceptance. This suggests that the significance of perception has a direct impact on user behavior^44^. In the context of autonomous driving systems, the importance attributed by users who engage with or experience these technologies reflects their perceived value. Generally, the more assistance these systems offer, the more substantial users consider their significance. Moreover, once users acknowledge the importance and value of these systems, they are more inclined to cultivate positive usage patterns^96^. Research indicates that user habits significantly affect loyalty and intentions to continue using the technology, underscoring the critical role of perceived importance in establishing and sustaining usage habits^97^. Consequently, users’ perception of importance plays a vital role in the development of behavioral habits.

#### **H7**

*There is a positive relationship between the perceived importance of assisted driving users and their driving habits.*

#### **H8**

*There is a positive relationship between the perceived importance of driverless users and their habits.*

The Expectation Confirmation Theory (ECT) posits that users’ ongoing intention to utilize a system is shaped by their satisfaction with it and their perceptions. This indicates that users who recognize their engagement in driving while utilizing the system and find their expectations fulfilled are more inclined to persist in its use^82^. Similarly, the Technology Acceptance Model (TAM) identifies perceived importance as a pivotal element affecting users’ acceptance and sustained utilization of technology^44^. Furthermore, established usage patterns and overall satisfaction can significantly contribute to the intention to continue employing the system^73,98^. Consequently, this paper asserts that, within the realm of autonomous driving systems, users’ perceived importance will enhance their intention to maintain usage of the system.

#### **H9**

*There is a positive relationship between the perceived importance of assisted driving users and their intention of continuous use.*

#### **H10**

*There is a positive relationship between the perceived importance of driverless users and their intention of continuous use.*

### User experience regret

When individuals recognize that an overlooked option could have led to a more favorable outcome than the one they chose, it triggers an uncomfortable sensation of regret, which is essential for the formation of experiential regret^99^. Regret theory suggests that when individuals perceive alternative choices as likely to produce better or worse results, they experience feelings of regret or relief^99^. Incorporating Kang’s viewpoint, the adverse emotional experience of regret following user interaction can negatively impact users^100^. As a result, users’ assessments of their experiences or interactions with autonomous driving systems may lean either positively or negatively. Positive evaluations foster psychological satisfaction, whereas negative assessments lead to feelings of regret. Thus, user satisfaction plays a critical role in influencing the intention to continue using the system^82^. Following user experiences, when expectations are met or surpassed, satisfaction with the system increases, thereby promoting the intention for ongoing use. Conversely, when expectations are not met, this intention diminishes^101^. The influence of experiential regret on the intention for continued usage is both necessary and significant. In essence, users who experience regret after engaging with the system may reduce or even cease their use of autonomous driving systems. The hypothesis presented in this paper is as follows:

#### **H11**

*There is a negative correlation between assisted driving user experience regret and continued use intent.*

#### **H12**

*There is a negative correlation between driverless user experience regret and continued use intent.*

Habits and behavioral intentions exhibit comparable automatic triggering mechanisms. When individuals encounter specific cues, this mechanism activates a corresponding behavioral response^102^. This “cue-response” relationship is rooted in previously rewarding behaviors. A positive experience associated with a behavior is crucial for habit formation, as such satisfaction enhances the likelihood of individuals consistently repeating those actions^103^. User experience, enjoyment, and cognitive absorption play significant roles in shaping usage intentions. Cognitive absorption is defined as the profound engagement users experience while interacting with technology. This state can bolster users’ intentions for continued use by augmenting perceived usefulness and ease of use^104^. Conversely, if users feel dissatisfied with their cognitive absorption after the experience, leading to feelings of regret, they may modify their existing usage patterns, thereby impeding habit formation^98^. Consequently, this paper proposes the following hypothesis:

#### **H13**

*There is a negative correlation between user experience regret and user driving habits.*

#### **H14**

*There is a negative correlation between driverless user experience regret and user driving habits.*

### User usage habit

Behavioral patterns typically emerge in consistent environments, characterized by similar physical and social contexts, through repetitive actions. Once these habitual behaviors are established, their execution no longer necessitates conscious reasoning; consequently, the more entrenched the habit, the less reliance there is on rational thought^103^. A prevalent conclusion in the field of social psychology is that previous habitual behaviors can serve as predictors of future actions, particularly when considering the impact of critical variables in rational action theory and the theory of planned behavior^102^. Related research indicates that habitual behaviors process information more swiftly and effortlessly than those requiring extensive deliberation, resulting in an increased frequency of consistent engagement with information technology. The findings also reveal a significant positive correlation between user habits and the intention to maintain usage^98^. Users are likely to cultivate habits through prolonged and frequent interaction with autonomous driving systems. These habits not only optimize time and enhance efficiency but also diminish cognitive load, evolving into automatic, spontaneous behaviors that affect the intention to continue usage. Therefore, this paper posits the following hypothesis:

#### **H15**

*There is a positive correlation between the driving habits of assisted driving users and their continuous use intention.*

#### **H16**

*There is a positive correlation between the driving habits of driverless users and the user’s continuous use intention.*

### The impact of autonomous driving related factors on continued use intent

To improve the predictive stability of the research model, this study integrates economic, safety, technological, ethical, social, and service factors as control variables. This methodology seeks to deepen the understanding of how antecedent and mediator variables within the research model influence the intention to continue utilizing autonomous driving systems. Notable progress has been achieved in energy management technology, battery efficiency, and intelligent driving algorithms within these systems, which not only enhance operational efficiency but also increase the economic advantages of vehicle usage^53^. The technical reliability of autonomous driving systems is paramount, as it dictates their capacity to make dependable real-time decisions^34^. These systems exhibit robust safety features across various domains, including pedestrian detection, obstacle recognition, and stereo matching^36,37^. After-sales and technical support pose significant challenges that impact users’ ongoing engagement, encompassing the management of intricate software systems, real-time data processing, and the complexities associated with experimental and road testing^63–65^. Additionally, users’ perceptions of ethical considerations are crucial in the deployment of autonomous driving systems, addressing concerns related to accident causation and prevention strategies, as well as the ethical dilemmas that arise during emergency decision-making^67,68^. Social influences also play a critical role; societal norms and the behaviors of others can profoundly impact an individual’s readiness to embrace new technologies^61^.

Therefore, automotive driver assistance systems, designed to aid users in operating vehicles, are influenced by various factors such as economic benefits, technological stability, safety capabilities, after-sales and technical services, ethical considerations, and social impacts, all of which affect users’ intentions for continued use.

#### **H17a**

*There is a positive correlation between economic benefits and users’ continuous intention to use.*

#### **H17b**

*There is a positive correlation between technological stability and users’ continuous intention to use.*

#### **H17c**

*There is a positive correlation between security capabilities and users’ continuous intention to use.*

#### **H17d**

*There is a positive correlation between after-sales and technical services and users’ continuous intention to use.*

#### **H17e**

*There is a positive correlation between ethical factors and users’ continuous intention to use.*

#### **H17f**

*There is a positive correlation between social influence and users’ continuous intention to use.*

Consequently, driverless systems, which replace the need for users to operate vehicles, are similarly influenced by economic benefits, technological stability, safety capabilities, after-sales and technical services, ethical considerations, and social impacts on users’ intentions for continued use.

#### **H18a**

*There is a positive correlation between economic benefits and users’ intentions for continued use.*

#### **H18b**

*There is a positive correlation between technological stability and users’ intentions for continued use.*

#### **H18c**

*There is a positive correlation between safety capabilities and users’ intentions for continued use.*

#### **H18d**

*There is a positive correlation between after-sales and technical services and users’ intentions for continued use.*

#### **H18e**

*There is a positive correlation between ethical considerations and users’ intentions for continued use.*

#### **H18f**

*There is a positive correlation between social impacts and users’ intentions for continued use.*

### Theoretical models

**Fig. 1:**
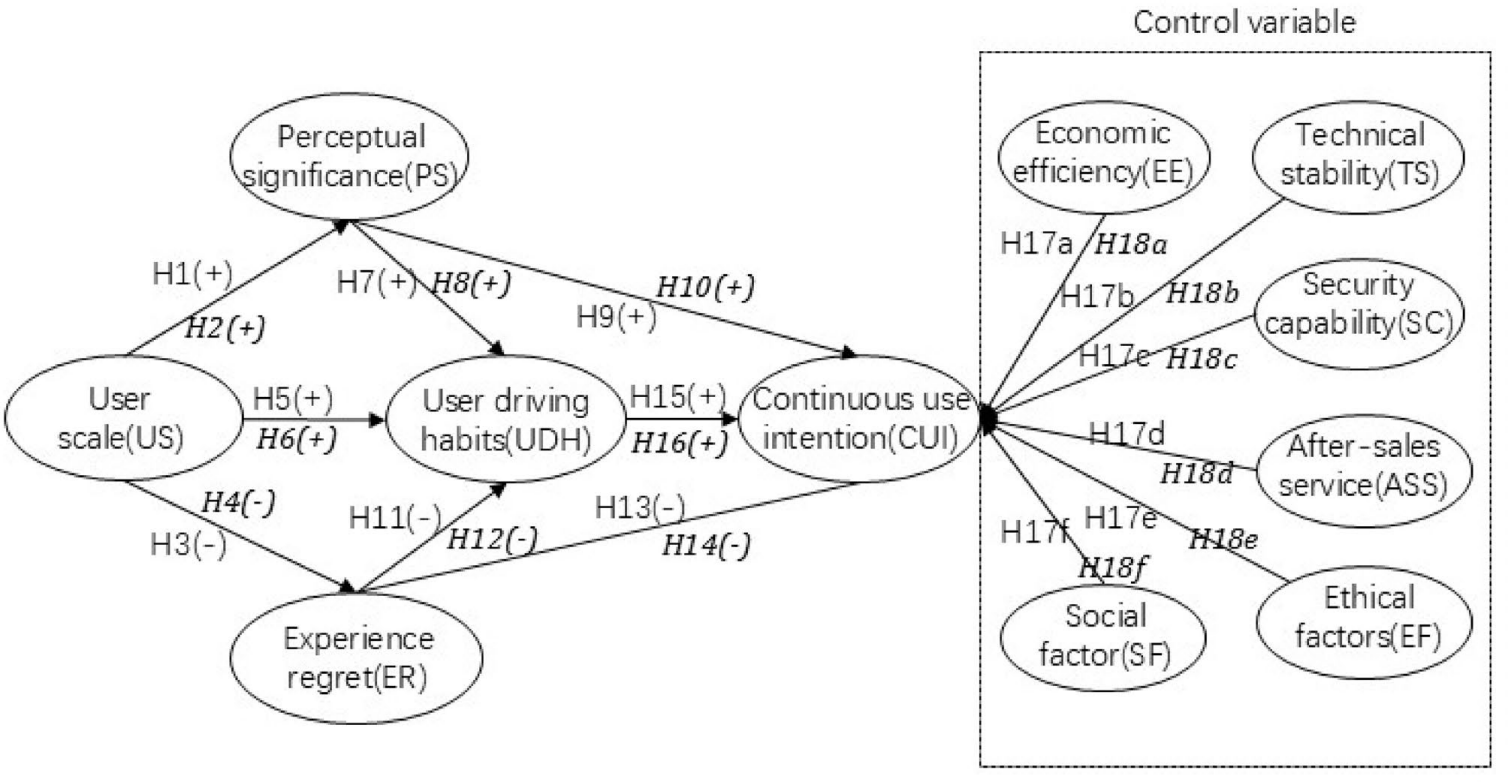
Structural equation model.

Based on the above theoretical analysis and hypothesis, this paper builds a research model, as shown in Fig. 1.

## Research framework, approaches and questionnaire design

### Research framework

**Fig. 2:**
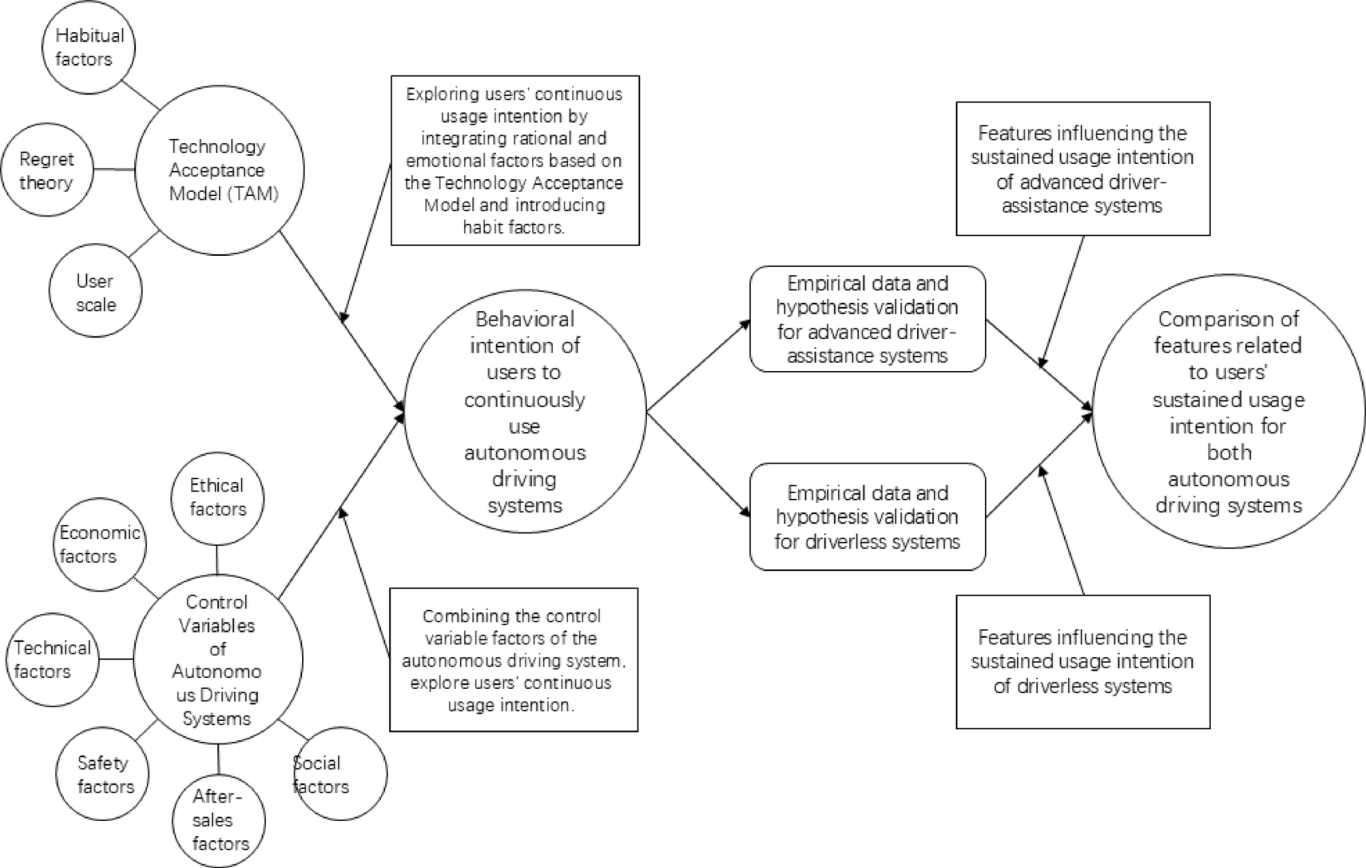
Organizational chart.

As illustrated in Fig. 2, the overall framework of this method is presented. This framework is divided into three key components: an extended theory of the Technology Acceptance Model (TAM), control variables for autonomous driving systems, and a comparative analysis of user continuous usage characteristics for two types of autonomous driving systems. Specifically, the first part focuses on the extended theory of the Technology Acceptance Model (TAM) to examine individual usage factors of consumers regarding autonomous driving systems. The second part investigates the relationship between control variables of autonomous driving systems and consumers’ continuous usage intention, including factors such as economics, technology, safety, ethics, society, and after-sales service. The third part involves data analysis and hypothesis validation to compare and analyze the conclusions regarding user continuous usage characteristics (i.e., individual factors and control variables) for the two autonomous driving systems.

## Research approaches

**Fig. 3:**
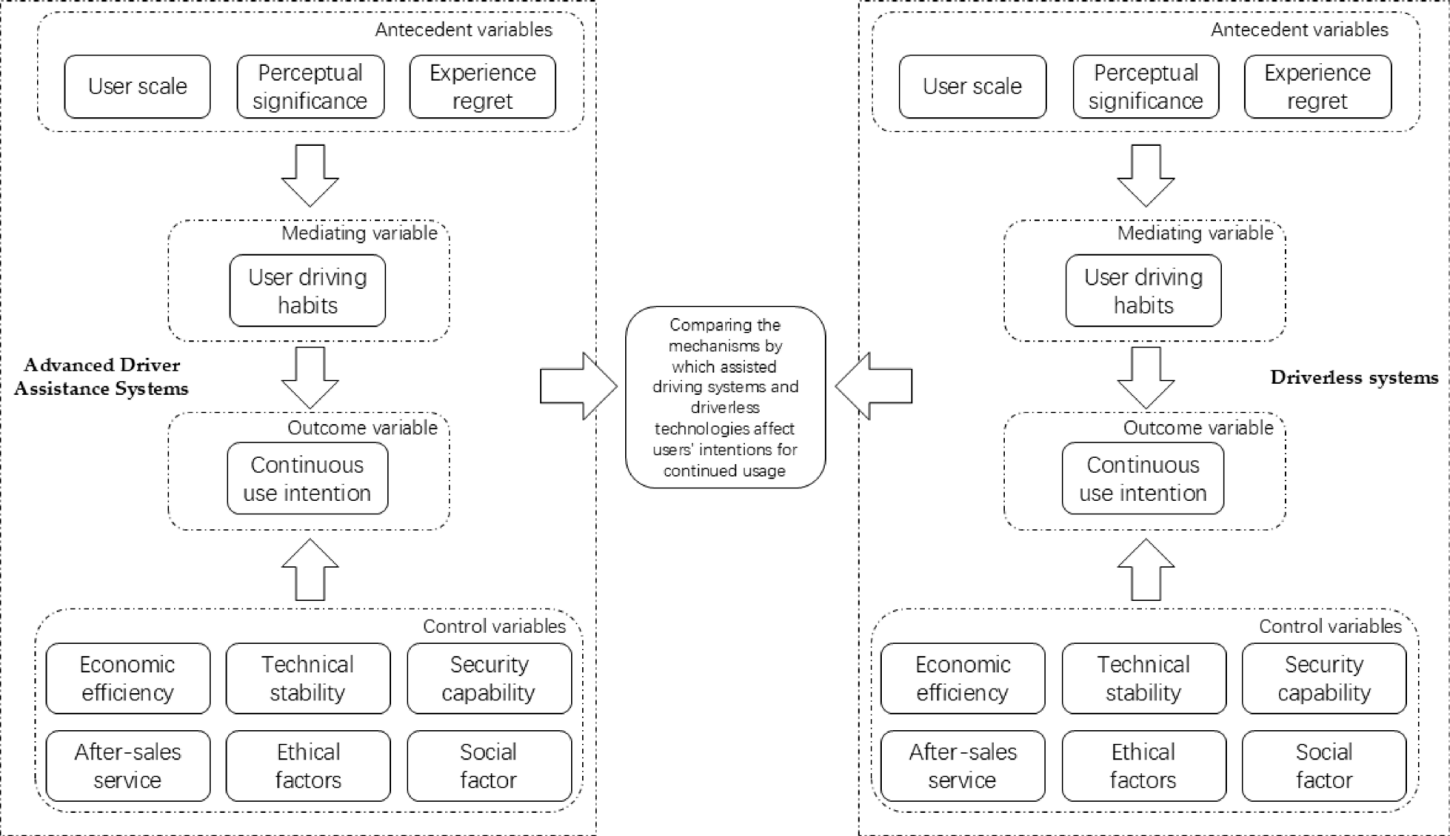
Research flow chart.

In terms of the research process, I began by reviewing literature both domestically and internationally on autonomous driving technology and user acceptance behavior. This comprehensive review allowed me to synthesize existing research findings, accurately identify research gaps, and define the direction and focus of this study, thereby establishing a solid theoretical foundation for subsequent research. Secondly, I conducted a comprehensive, multi-level, and systematic comparison of advanced driver-assistance systems (ADAS) and driverless systems, focusing on control variables and user experience. This in-depth analysis aimed to uncover the differences between the two systems and how these differences influence user intention, providing a unique perspective and deep insights for the research. Based on this, as shown in Fig. 3, I constructed a structural equation model (SEM) to analyze users’ continuous usage intention of autonomous driving systems. The intuitive flowchart clearly illustrates the model’s variable composition and logical relationships: antecedent variables influence the outcome variable (continuous usage intention) through a mediating variable (habit), with control variables integrated as external moderating factors within the entire model, reflecting a dual-dimensional driving mechanism of rational and non-rational factors. Furthermore, through the comparison of the two autonomous driving systems, I identified specific differentiated characteristics that affect users’ continuous usage intention.

Simultaneously, we collected data through a survey, targeting two distinct user groups: those utilizing ADAS and those experienced with driverless systems. Specifically, both groups completed the same questionnaire, with clear instructions to indicate the type of driving system they had used or experienced. The application of identical questionnaires and the SEM allowed us to effectively compare the differential impacts of the two autonomous driving systems on users, reflecting their varied understanding of the usage characteristics of each system type. This comparative analysis focused on identifying and contrasting the differentiating features between the two systems.

Although driverless systems represent an inevitable trend in the future development of automotive driving systems, current driver assistance systems remain the mainstream autonomous driving systems in the market. During this critical transitional period in the development of automotive driving systems, it is particularly important to drive innovation in autonomous driving systems based on user usage patterns. This is a pivotal phase concerning the service of technology to humanity. The findings of this research will not only provide a certain data reference for the future development of autonomous driving systems and the study of segmented systems but will also aid in clarifying the future direction of autonomous driving system development through mathematical analysis.

### Questionnaire design

This study utilizes scales derived from the literature of relevant scholars, which have been widely applied. Based on the specific research content, the scales have been contextually adjusted. In light of this, relevant questionnaires were designed in conjunction with the aforementioned literature, aiming to validate the mechanisms and effects of both advanced driver-assistance systems (ADAS) and driverless systems on consumer perceptions of importance, experience of regret, user driving habits, and intentions for continued use. Additionally, the study explores the impact of control variables, including economic, technological, safety, ethical, social, and after-sales factors, on users’ intentions for continued use. The research will validate users’ intentions for continued use from two dimensions, with the expectation of providing a reference for the improvement and development of ADAS and autonomous driving systems, thereby promoting their further advancement and offering insights for the future development of autonomous driving systems.

The scale measuring user size incorporates content from Lee^92^ and Karapanos^91^, utilizing items (US1-US3) for measurement. For the latent variables of perceived importance and experience of regret, the scale adaptation is based on Davis^44^, Yaffe^96^, and Kim^97^, employing items (PS1-PS3). The content related to experience satisfaction and regret is adapted from Choi^101^, Lankton^98^, and Kang^100^, measured by items (ER1-ER3). User driving habits are derived from Aarts^103^ and assessed using items (UDH1-UDH3). Adaptations regarding continuous usage intentions are sourced from Agarwal^103^ and Limayem^73^, employing items (CUI1-CUI3) for measurement.

The scale for economic benefits among the control variables is adapted from the relevant content of Vasebi^53^ and measured using items (EE1-EE3). The scale for technical stability is derived from Liu^34^ and measured with items (TS1-TS3). The scale for safety capability is adapted from the works of Krizhevsky^36^ and Simonyan^37^, utilizing items (SC1-SC3). The scale for after-sales and technical service is based on the content from Beringhoff^63^, Costache^64^, and Koopman^65^, measured with items (ASS1-ASS3). The ethical factors scale is adapted from the relevant content of Robinson^67^ and Bonnefon^68^, utilizing items (EF1-EF3). The scale for social influence is based on the relevant content from Jing^61^ and measured using items (SF1-SF3). The questionnaire design items are summarized in Table 2.

**Table 2:** Definition of Variable Operability and Reference Scales.

Construct	Items	Source
User scale (US)	User acceptance of autonomous driving systems is positioned as the mass acceptance of two different types of autonomous driving systems. This includes many people using it, friends around it, and so on	^91,92^
Perceptual significance (PS)	The importance of the user to the autonomous driving system is positioned as the perceived importance of the user to the two different autonomous driving systems. These include the importance of use, the relevance and indispensability of life	^44,96,97^
Experience regret (ER)	The user’s bad experience of the automatic driving system is positioned as the user’s bad experience of the two different automatic driving systems. These include experience regret, use regret, and refusal to use	^98,100,101^
User driving habits (UDH)	The user’s dependence on the use of automatic driving system is positioned as the user’s driving habit in the use of two different driving systems. These include approval systems, common use and first choice	^103^
Economic efficiency (EE)	User perceptions of economic savings from autonomous driving systems are positioned as savings for users from two different types of autonomous driving systems. These include saving car costs and improving energy efficiency	^53^
Technical stability (TS)	Users’ views on the technical reliability of autonomous driving systems are positioned as the technical reliability provided to users by two different types of autonomous driving systems. This includes the stability and protection of system technology	^34^
Security capability (SC)	Users’ views on the safety capabilities of autonomous driving systems are positioned as two different types of autonomous driving systems providing safety services to users. These include protection of driving safety, prediction of driving risks and emergency judgment and treatment	^36,37^
After-sales service (ASS)	The user’s view of the after-sales service ability of the automatic driving system is positioned as the impact of the after-sales service ability provided by the two different types of automatic driving system on the user’s use. These include the durability of system maintenance, the trust of maintenance assurance and personalized assurance service	^63–65^
Ethical factors (EF)	Users’ views on the ethical aspects of autonomous driving systems are positioned as the ethical and moral services provided to users by two different types of autonomous driving systems. Among them, humanized treatment of special circumstances, in line with social ethics, and humane treatment of problems	^67,68^
Social factor (SF)	Users’ views on the social factors of autonomous driving systems are positioned as the social factors that two different types of autonomous driving systems bring to users. Among them, the influence of social environment and surrounding people	^61^
Continuous use intention (CUI)	The user’s view of the continuous use intention of the autonomous driving system is positioned as the continuous use brought by the two different types of autonomous driving system to the user. These include that the user has been using such autonomous driving systems, that the user will use such autonomous driving systems in the future, and recommend others to use them	^73,104^

## Informed consent

Given the practical considerations of surveying users of two distinct driver assistance systems, we opted for an online questionnaire methodology. The geographically dispersed nature of users of automotive driver assistance systems renders offline surveys inefficient for comprehensive coverage. To ensure data authenticity and validity, an online survey was deemed the superior approach. Furthermore, online surveys leverage internet platforms to transcend geographical limitations, enabling access to users from diverse regions and backgrounds. This contrasts with offline surveys, offering a broader sample pool and enhancing the generalizability of our findings. Participants completing the questionnaire in familiar environments can more accurately recall and describe their experiences with the driver assistance systems, mitigating behavioral biases that might arise in controlled experimental settings. This approach ensures that the research outcomes more closely reflect real-world usage scenarios, thereby increasing their applicability. Considering that driverless systems are currently primarily deployed in the Robotaxis sector and are not yet widely adopted in the consumer market, and given that pilot operations of Robotaxis are underway in cities such as Shanghai, Beijing, and Guangzhou in China, the online survey method allows for precise targeting of the intended user base. Consequently, all respondents were recruited online. Moreover, this study has received ethical approval from the School of Fine Arts at Nanjing Normal University. Prior to commencing the survey, participants were presented with an online informed consent form. Only after providing verbal consent and selecting the “agree” option were they permitted to proceed to the questionnaire. Participants who selected the “disagree” option were immediately excluded from the survey. Given the nature of the research, which pertains to automotive driving and usage, minors were excluded from participation.

Participants were recruited via the Wenjuanxing online survey platform, leveraging social media platforms such as WeChat to facilitate survey distribution. Given the study’s focus on automotive driving and usage, eligibility was restricted to individuals aged 18 years or older to ensure adequate cognitive capacity and comprehension for accurate questionnaire completion and valid feedback. Specifically, users of advanced driver-assistance systems (ADAS) were required to have practical experience with ADAS features (e.g., adaptive cruise control, lane-keeping assist). Conversely, participants evaluating driverless vehicle systems were required to have prior experience riding in driverless taxis (Robotaxis). This criterion ensured that participants possessed direct experience with the driving systems under investigation, thereby enabling the collection of authentic and reliable data.

To ensure the online sample effectively represented the broader demographic characteristics of ADAS and fully autonomous vehicle users, a stratified quota sampling approach was implemented. Stratification was based on factors such as the geographic distribution of Robotaxis pilot cities and ADAS users, as well as age and gender. For instance, Robotaxis pilot cities were categorized into regional strata (e.g., Shanghai, Beijing, Guangzhou). Further stratification was applied across age cohorts (e.g., 18–24 years, 24–35 years) and gender (male, female), ensuring proportional representation within each stratum to encompass diverse user backgrounds. This approach aimed to align the sample’s age, gender, and other demographic characteristics with the overall population structure. Through these methodologies, the selected online sample exhibited demographic similarities in key characteristics (age, gender, region) to the general population of ADAS and fully autonomous vehicle users, thereby providing a representative basis for the study and supporting the generation of reliable data.

Furthermore, this study has received ethical approval from the School of Fine Arts at Nanjing Normal University. Prior to the commencement of the questionnaire survey, an online informed consent form will be presented. Participants will proceed to the questionnaire only after providing verbal consent and selecting the “agree” option. Selecting the “decline” option will immediately terminate the survey. Given the study’s focus on automobile driving and usage, participation is restricted to adults, excluding minors.

## Empirical study 1: research on the continuous use intention of the users of the driver assistance system

### Sample demographic analysis

This study distributed questionnaires to users of vehicles equipped with advanced driver-assistance systems and to users of driverless taxis through an online platform. In addition to basic personal information, all items were rated using a Likert scale ranging from 1 (strongly disagree) to 7 (strongly agree). All respondents voluntarily answered the questions with informed consent and were free to withdraw from the survey at any time.

The design of the current questionnaire survey is divided into three sections. Initially, we collected basic demographic information, including age, gender, occupation, and educational attainment. Subsequently, we inquired about the type of driving system participants engaged with or experienced, along with the frequency of their participation. Finally, the questionnaire incorporated specialized items pertinent to the study’s objectives. To ensure data quality, several standards were implemented during the survey administration. Each respondent was limited to a single participation, and the minimum completion time was set at three minutes. Furthermore, responses containing more than five identical or contradictory answers were excluded. Consequently, a total of 600 questionnaires were distributed, yielding 503 valid responses after applying the aforementioned screening criteria, resulting in an effective response rate of 83.8%.

This study collected a valid sample of 503 users of automotive driver assistance systems. The questionnaire comprised 33 questions, with valid responses totaling 503, meeting Jackson’s criterion of a parameter-to-sample size ratio exceeding 1:10^105^. Consequently, the data analysis was conducted based on this standard, utilizing software such as SPSS 22.0 and Smart PLS 4.0 for data processing. As shown in Table 3, a descriptive statistical analysis of the demographic variables of the user sample for automotive driver assistance systems is presented.

**Table 3:** Demographic profile of sample (n = 503).

Sample	Category	Number	Percentage (%)
Gender	Male	284	56.5
	Female	219	43.5
Age	18–24	58	11.5
	25–34	128	25.4
	35–44	204	40.6
	45–54	96	19.1
	55–70	17	3.4
Occupation	Student	36	7.2
	Freelance or self-employed	381	75.7
	Public officials or public institutions	49	9.7
	Others	37	7.4
Ratings using the autopilot system	Use multiple times a day	18	3.6
	Use once a day	102	20.3
	Use once a week	179	35.6
	Use it once or twice a month	142	28.2
	Use less than or equal to once a month	62	12.3

Based on the results of the questionnaire survey regarding advanced driver-assistance systems (ADAS), it is evident that the male participants slightly outnumber the female participants. In terms of age distribution, the majority of respondents fall within the 25 to 44-year-old demographic, representing a young to middle-aged cohort. Regarding occupational distribution, freelancers constitute a significant proportion across all professional categories. In terms of usage frequency, users of ADAS predominantly utilize the system once a day or once a week, with instances of multiple uses within a single day being relatively rare. Given the actual usage patterns of household vehicles in contemporary society, the likelihood of multiple daily uses is inherently low. Therefore, the sample proportions across various sections of this survey are reasonably balanced and meet the research requirements. Considering the current age distribution and the findings on usage frequency, it is clear that conducting this research on advanced driver-assistance systems is highly necessary. The outcomes of this study can provide valuable insights and constructive recommendations for companies focused on the development and enhancement of ADAS functionalities within autonomous driving systems.

### Reliability and validity analysis

Reliability pertains to the degree to which a measurement instrument can yield consistent outcomes upon repeated applications. This includes aspects such as measurement precision, consistency, and resilience^106^. The Cronbach’s alpha coefficients for various measurement variables were computed utilizing SPSS 22.0 software. A coefficient exceeding 0.6 signifies that the data within the scale demonstrates both accuracy and validity^107^. As illustrated in Table 4, all Cronbach’s alpha coefficients derived from the survey data were above 0.7. Furthermore, the total correlation following the removal of items remained above 0.5, and the Cronbach’s alpha coefficients after the exclusion of any item did not fall below the current results. This indicates that the items should remain intact, suggesting that the scale employed in this study exhibits strong reliability.

**Table 4:** Reliability analysis results of driving assistance system (*n* = 503).

Dimension	Items	Corrected item-to-total correlation	Cronbach’s α if item deleted	Cronbach’s α
US	US1	0.704	0.786	0.843
	US2	0.713	0.778	
	US3	0.709	0.782	
PS	PS1	0.620	0.723	0.789
	PS2	0.627	0.716	
	PS3	0.640	0.701	
ER	ER1	0.593	0.722	0.777
	ER2	0.615	0.697	
	ER3	0.632	0.679	
UDH	UDH1	0.673	0.755	0.821
	UDH2	0.681	0.746	
	UDH3	0.671	0.757	
EE	EE1	0.608	0.723	0.784
	EE2	0.626	0.704	
	EE3	0.633	0.696	
TS	TS1	0.623	0.714	0.787
	TS2	0.633	0.703	
	TS3	0.622	0.715	
SC	SC1	0.626	0.741	0.789
	SC2	0.652	0.714	
	SC3	0.647	0.718	
ASS	ASS1	0.646	0.737	0.804
	ASS2	0.632	0.752	
	ASS3	0.673	0.708	
EF	EF1	0.672	0.792	0.834
	EF2	0.710	0.754	
	EF3	0.701	0.763	
SF	SF1	0.661	0.733	0.808
	SF2	0.643	0.751	
	SF3	0.667	0.727	
CUI	CUI1	0.668	0.753	0.818
	CUI2	0.660	0.761	
	CUI3	0.684	0.736	

Harman’s single-factor test is a widely used method for assessing common method bias (CMB). If CMB is present, all variables will load onto a single factor in an unrotated factor analysis, or a single factor will account for a substantial proportion of the variance^108^. Podsakoff and Organ suggest that if a single factor accounts for less than 50% of the variance in an unrotated exploratory factor analysis (EFA), CMB is likely within acceptable limits^109^. To examine for common method bias in this study, we employed Harman’s single-factor test using SPSS. As shown in Table 5, the results of the EFA revealed five factors with eigenvalues greater than 1, cumulatively explaining 59.654% of the variance. The first unrotated factor accounted for 45.177% of the variance, which is below the 50% threshold. This indicates that no single factor explains the majority of the variance, suggesting that common method bias is well-controlled in this study.

**Table 5:** Common variance explanation of driving assistance system (*n* = 503).

Element	Initial eigenvalue			Extract the sum of squares and load		
	Aggregate	Variable (%)	Cumulative (%)	Aggregate	Variable (%)	Cumulative (%)
1	14.908	45.177	45.177	14.908	45.177	45.177
2	1.377	4.172	49.350	1.377	4.172	49.350
3	1.244	3.769	53.118	1.244	3.769	53.118
4	1.099	3.331	56.450	1.099	3.331	56.450
5	1.058	3.205	59.654	1.058	3.205	59.654
6	0.993	3.008	62.662			
7	0.963	2.918	65.581			
8	0.839	2.541	68.122			
9	0.699	2.118	70.240			
10	0.593	1.797	72.037			
11	0.539	1.634	73.670			
12	0.534	1.617	75.287			
13	0.515	1.562	76.849			
14	0.506	1.534	78.384			
15	0.496	1.504	79.888			
16	0.477	1.446	81.334			
17	0.462	1.400	82.734			
18	0.454	1.377	84.111			
19	0.442	1.338	85.449			
20	0.427	1.295	86.745			
21	0.415	1.258	88.002			
22	0.401	1.214	89.217			
23	0.384	1.162	90.379			
24	0.369	1.119	91.497			
25	0.361	1.093	92.591			
26	0.354	1.074	93.665			
27	0.339	1.026	94.691			
28	0.325	0.985	95.675			
29	0.314	0.951	96.626			
30	0.306	0.926	97.552			
31	0.295	0.895	98.447			
32	0.263	0.797	99.244			
33	0.249	0.756	100.000			

Exploratory factor analysis was conducted using SPSS 22.0, employing the Kaiser–Meyer–Olkin (KMO) measure and Bartlett’s test of sphericity to ascertain sufficient correlations for factor extraction^110^. The preliminary analyses indicated that the data were suitable for factor analysis. Specifically, the Kaiser–Meyer–Olkin (KMO) values exceeded the 0.5 threshold, and the Bartlett’s Test of Sphericity yielded statistically significant results (*p* < 0.05) for all variables, indicating that the data met the requirements for factor analysis^111,112^. Furthermore, the extraction of a single factor with an eigenvalue greater than 1 for each variable, coupled with cumulative variance contributions exceeding 50%, suggested that the extracted factors effectively explained the variance in the variables. The commonalities for all items were above 0.5, and the factor loadings were above 0.6, indicating a strong unidimensionality^113^. As presented in Table 6, the KMO values ranged from 0.701 to 0.729, all exceeding the 0.5 threshold, and the Bartlett’s Test of Sphericity was significant (*p* < 0.05) for all variables, confirming the suitability of the data for factor analysis. Subsequently, a principal component analysis was conducted. The results revealed that each variable extracted only one factor with an eigenvalue greater than 1, and the cumulative variance contribution for each variable was at least 69.845%, exceeding the 50% threshold. This suggests that the factors derived from the two scales in this study effectively explained the variables. The commonalities for all items were at least 0.683, exceeding 0.5, and the factor loadings were at least 0.826, exceeding 0.6, all within the recommended range. Therefore, the survey results of this study demonstrated robust unidimensionality.

**Table 6:** Exploratory factor analysis results of driving assistance system (*n* = 503).

Dimension	Items	KMO	Bartlett sphere test	Factor loading	Commonality	Eigenvalue	Total variation explained (%)
US	US1	0.729	0	0.870	0.757	2.285	76.151
	US2			0.875	0.766		
	US3			0.873	0.761		
PS	PS1	0.707	0	0.833	0.693	2.109	70.307
	PS2			0.837	0.700		
	PS3			0.846	0.716		
ER	ER1	0.701	0	0.818	0.669	2.076	69.207
	ER2			0.834	0.695		
	ER3			0.844	0.712		
UDH	UDH1	0.720	0	0.857	0.734	2.209	73.632
	UDH2			0.862	0.743		
	UDH3			0.855	0.732		
EE	EE1	0.704	0	0.826	0.683	2.095	69.845
	EE2			0.838	0.703		
	EE3			0.843	0.710		
TS	TS1	0.705	0	0.843	0.710	2.097	69.897
	TS2			0.832	0.693		
	TS3			0.833	0.694		
SC	SC1	0.712	0	0.844	0.713	2.145	71.512
	SC2			0.850	0.722		
	SC3			0.843	0.711		
ASS	ASS1	0.713	0	0.846	0.716	2.155	71.841
	ASS2			0.847	0.718		
	ASS3			0.849	0.721		
EF	EF1	0.716	0	0.845	0.714	2.216	73.862
	EF2			0.857	0.735		
	EF3			0.876	0.767		
SF	SF1	0.712	0	0.854	0.729	2.157	71.891
	SF2			0.836	0.698		
	SF3			0.854	0.729		
CUI	CUI1	0.716	0	0.849	0.722	2.184	72.808
	CUI2			0.848	0.719		
	CUI3			0.863	0.744		

Using AMOS for confirmatory factor analysis, the factor loadings of each item in the scale were found to be greater than 0.5, indicating that all items consistently explain their respective variables. This consistency not only suggests that the items effectively reflect the essence of the variables but also demonstrates that the scale possesses high stability and reliability in measuring these variables^114^. The composite reliability (CR) and average variance extracted (AVE) are crucial metrics for assessing the convergent validity of related variables, as indicated by relevant research. Specifically, the CR value should not fall below 0.7, and the AVE value should not be less than 0.5^115,116^. In accordance with Table 7, the CR and AVE were calculated based on the factor loadings of each item. The factor loadings in this study exceeded 0.5, and the CR values were all above 0.778. Furthermore, the square root of the AVE exceeded 0.539, surpassing the thresholds of 0.7 for CR and 0.5 for AVE. These findings suggest that the related variables demonstrate robust convergent validity.

**Table 7:** Convergent factor analysis results of driving assistance system (*n* = 503).

Dimension	Items	Unstandardized factor loading	Standardize factor loading	SE	*P*-Value	AVE	CR
US	US1	1	0.815	–	–	0.642	0.843
	US2	1.019	0.797	0.049	0		
	US3	0.989	0.792	0.048	0		
PS	PS1	1	0.750	–	–	0.554	0.789
	PS2	0.994	0.743	0.056	0		
	PS3	1.022	0.740	0.057	0		
ER	ER1	1	0.706	–	–	0.539	0.778
	ER2	1.043	0.748	0.063	0		
	ER3	1.030	0.748	0.062	0		
UDH	UDH1	1	0.789	–	–	0.603	0.820
	UDH2	0.978	0.746	0.052	0		
	UDH3	1.086	0.793	0.053	0		
EE	EE1	1	0.720	–	–	0.549	0.785
	EE2	1.030	0.734	0.061	0		
	EE3	1.076	0.767	0.061	0		
TS	TS1	1	0.763	–	–	0.550	0.786
	TS2	0.913	0.717	0.053	0		
	TS3	0.954	0.744	0.054	0		
SC	SC1	1	0.751	–	–	0.568	0.798
	SC2	0.999	0.759	0.055	0		
	SC3	1.027	0.751	0.057	0		
ASS	ASS1	1	0.756	–	–	0.578	0.804
	ASS2	1.024	0.756	0.056	0		
	ASS3	1.034	0.769	0.056	0		
EF	EF1	1	0.784	–	–	0.626	0.834
	EF2	1.017	0.804	0.050	0		
	EF3	0.988	0.786	0.050	0		
SF	SF1	1	0.769	–	–	0.585	0.809
	SF2	0.973	0.756	0.053	0		
	SF3	1.061	0.769	0.056	0		
CUI	CUI1	1	0.772	–	–	0.600	0.818
	CUI2	0.997	0.770	0.053	0		
	CUI3	0.990	0.782	0.056	0		

0.5 is the minimum standard for AVE, and CR ≥ 0.7

Employing the Fornell-Larcker criterion, discriminant validity assesses the degree of differentiation among latent variables. If the square root of the Average Variance Extracted (AVE) for all latent variables does not exceed 0.9, the scale demonstrates satisfactory discriminant validity^116^. Within the framework of Structural Equation Modeling (SEM), the assessment of correlations among exogenous constructs, which serve as antecedent factors influencing other variables, is crucial for examining discriminant validity. Research suggests that when the correlation coefficients between exogenous constructs are below 0.85, the model effectively distinguishes between different exogenous variables, thereby mitigating the potential for multicollinearity to compromise the accuracy of research findings^117^.

As demonstrated in Table 8, the square root of the Average Variance Extracted (AVE) for each latent variable exceeds the correlation coefficients among the corresponding latent variables. This indicates that the primary variables exhibit significant associations while maintaining sufficient discriminant validity, and all correlation coefficients are below 0.9. Furthermore, the correlation coefficients between exogenous constructs in this study are all below 0.85. This finding not only aligns with the discriminant validity criteria proposed by relevant scholars but also further validates that the two tested models possess ideal discriminant validity. This suggests that the research model can clearly define different constructs, effectively avoiding confusion between variables, and ensuring the scientific rigor of the research measurement tools, thereby providing a reliable foundation for subsequent data analysis and theoretical validation.

**Table 8:** Correlation coefficient and average extraction variance of the assisted driving system (*n* = 503).

Latent variable	ASS	EE	EF	SC	SF	TS	ER	PS	CUI	US	UDH
ASS	0.848										
EE	0.806	0.836									
EF	0.802	0.822	0.866								
SC	0.814	0.813	0.813	0.844							
SF	0.805	0.809	0.814	0.803	0.85						
TS	0.780	0.801	0.799	0.789	0.788	0.837					
ER	− 0.805	− 0.798	− 0.78	− 0.795	− 0.774	− 0.776	0.832				
PS	0.818	0.814	0.796	0.795	0.814	0.791	− 0.791	0.838			
CUI	0.784	0.811	0.827	0.792	0.808	0.800	− 0.78	0.821	0.856		
US	0.808	0.816	0.817	0.807	0.809	0.784	− 0.786	0.808	0.807	0.873	
UDH	0.810	0.818	0.819	0.812	0.822	0.794	− 0.805	0.820	0.829	0.827	0.858

### Model testing

Research indicates that the SRMR value is one of the indices used to assess model fit; a smaller SRMR value signifies a better fit of the model to the data. When the SRMR value is below 0.08, it suggests that the model fits the data well^118,119^. A d-ULS value of less than 0.95 indicates a good fit of the model to the data, with a moderate number of free parameters^120^. Similarly, a d-G value below 0.95 also suggests a good fit of the model to the data, along with a reasonable number of free parameters^121^. Furthermore, an NFI greater than 0.8 indicates a high model fit, with values closer to 1 reflecting a stronger fit, demonstrating the model’s ability to accurately capture the structures and relationships within the data^122^. Therefore, as shown in Table 9, the SRMR value of 0.039 is below the threshold of 0.08, the d-ULS value of 0.852 and the d-G value of 0.731 are both below the threshold of 0.95, and the NFI value of 0.844 exceeds 0.8, indicating that the model of the driver assistance system fits the data well.

**Table 9:** The model fitting measure of the assisted driving system.

Common indices	d-ULS	d-G	SRMR	NFI
Criteria	< 0.95	< 0.95	< 0.08	> 0.8
Values	0.852	0.731	0.039	0.844

### Path hypothesis analysis

Using PLS-SEM to test hypotheses and calculate path coefficients, I employed the Bootstrap method with 5,000 resamples. The research values were below 0.05, indicating statistical significance. Therefore, as seen in Table 8, the path coefficient test results for the user’s continuous usage intention of the driver assistance system show that user scale has a positive significant impact on perceived importance (T = 58.778, *P* < 0.05), regret after experience (T = 48.483, *P* < 0.05), and user driving habits (T = 9.059, *P* < 0.05), supporting hypotheses H1/H3/H5. Additionally, perceived importance positively significantly affects user driving habits (T = 8.077, *P* < 0.05) and continuous usage intention (T = 4.283, *P* < 0.05), supporting hypotheses H7/H9. Regret after experience negatively significantly impacts user driving habits (T = 9.281, *P* < 0.05), supporting H11. However, it has no significant negative correlation with continuous usage intention, thus hypothesis H13 is not supported. User driving habits positively significantly influence continuous usage intention (T = 4.283, *P* < 0.05), supporting hypothesis H15. The results also indicate that H17a economic benefits (T = 2.027, *P* < 0.05), H17b technological stability (T = 3.089, *P* < 0.05), H17e ethical factors (T = 4.433, *P* < 0.05), and H17f. social factors (T = 2.233, *P* < 0.05) directly affect users’ continuous usage intention, while H17c safety capability (T = 0.819, *P* > 0.05) and H17d after-sales service (T = 0.157, *P* > 0.05) do not have a direct impact. As summarized in Table 10, the results of the hypothesis tests are presented.

**Table 10:** The test of the path relation of the hypothesis model of the assisted driving system.

Hypothesis	Relationship	Coefficient	T statistics	*P* values	Results
H1	US—> PS	0.808	58.778	0	Accept
H3	US—> ER	-0.786	48.438	0	Accept
H5	US—> UDH	0.357	9.059	0	Accept
H7	PS—> UDH	0.313	8.077	0	Accept
H9	PS—> CUI	0.186	4.283	0	Accept
H11	ER—> UDH	-0.276	9.281	0	Accept
H13	ER—> CUI	-0.052	1.191	0.234	Not accept
H15	UDH—> CUI	0.185	4.283	0	Accept
H17a	EE—> CUI	0.094	2.027	0.043	Accept
H17b	TS—> CUI	0.125	3.089	0.002	Accept
H17c	SC—> CUI	0.040	0.878	0.380	Not accept
H17d	ASS—> CUI	-0.007	0.157	0.875	Not accept
H17e	EF—> CUI	0.203	4.433	0	Accept
H17f.	SF—> CUI	0.098	2.233	0.026	Accept

**Fig. 4:**
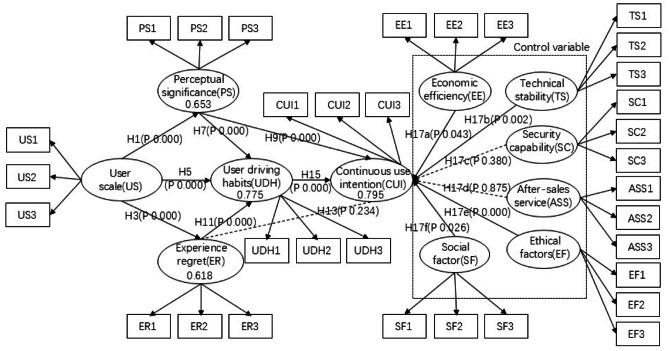
Hypothesis test results of the assisted driving system.

According to the significant hypothesis testing results presented in the table, as shown in Fig. 4

## Empirical study 2: continuous user intent research for driverless system

### Sample demographic analysis

This study distributed questionnaires to users of vehicles equipped with advanced driver-assistance systems and to users of driverless taxis through an online platform. In addition to basic personal information, all items were rated using a Likert scale ranging from 1 (strongly disagree) to 7 (strongly agree). All respondents voluntarily answered the questions with informed consent and were free to withdraw from the survey at any time.

The design of the current questionnaire survey is divided into three sections. Initially, we collected basic demographic information, including age, gender, occupation, and educational attainment. Subsequently, participants were asked to specify the type of driving system they engaged with, whether through direct operation or experiential interaction, along with the frequency of their participation. Finally, the questionnaire incorporated specialized items pertinent to the study’s objectives. To ensure data integrity, several quality control measures were implemented. Each respondent was limited to a single participation, and a minimum completion time of three minutes was enforced. Furthermore, responses exhibiting more than five identical or contradictory answers were excluded. Consequently, a total of 600 questionnaires were distributed, yielding 504 valid responses after applying the aforementioned screening criteria, resulting in an effective response rate of 84%.

This study collected a valid sample of 504 users of driverless systems. The questionnaire comprised 33 questions, with 504 valid responses, meeting Jackson’s criterion of a parameter-to-sample size ratio exceeding 1:10^105^. Consequently, the data analysis was conducted based on this standard, utilizing software such as SPSS 22.0 and Smart PLS 4.0 for processing. As shown in Table 11, a descriptive statistical analysis of the demographic variables of the sample population of users of driverless taxi systems is presented.

**Table 11:** Demographic profile of sample (*n* = 504).

Sample	Category	Number	Percentage (%)
Gender	Male	297	58.9
	Female	207	41.1
Age	18–24	71	14.1
	25–34	129	25.6
	35–44	173	34.3
	45–54	89	17.7
	55–70	42	8.3
Occupation	Student	40	7.9
	Freelance or self-employed	362	71.8
	Public officials or public institutions	47	9.3
	Others	55	10.9
Ratings using the autopilot system	Use multiple times a day	41	8.1
	Use once a day	88	17.5
	Use once a week	196	38.9
	Use it once or twice a month	141	28
	Use less than or equal to once a month	38	7.5

The survey on driverless systems indicates that the proportion of male respondents is slightly higher than that of female respondents. The age distribution predominantly features young and middle-aged individuals between 25 and 44 years old. In terms of occupational distribution, freelancers represent a significant portion. Analyzing the data from the questionnaires for both assisted driving systems and driverless systems reveals that user engagement is primarily concentrated around daily and weekly usage frequencies. The frequency of multiple daily uses for assisted driving systems is lower than that for users of driverless systems. Based on observations in real-life scenarios, it is evident that the usage frequency of autonomous taxis can indeed surpass that of personal vehicles. Therefore, the sample proportions across various sections of the survey are reasonable and meet the research requirements. Given the age distribution and usage frequency, this comparative study on driverless systems is essential, as it can provide valuable insights and recommendations for companies looking to enhance the development of driverless systems.

### Reliability and validity analysis

Reliability pertains to the degree to which a measurement instrument can yield consistent outcomes upon repeated applications. This includes aspects such as measurement precision, consistency, and resilience^106^. The Cronbach’s alpha coefficients for various measurement variables were computed utilizing SPSS 22.0 software. A coefficient exceeding 0.6 signifies that the data within the scale demonstrates both accuracy and validity^107^. As illustrated in Table 12, all Cronbach’s alpha coefficients derived from the survey data were above 0.7. Furthermore, the total correlation following the removal of items remained above 0.5, and the Cronbach’s alpha coefficients after the exclusion of any item did not fall below the current results. This indicates that the items should remain intact, suggesting that the scale employed in this study exhibits strong reliability.

**Table 12:** Reliability analysis results of driverless system (*n* = 504).

Dimension	Items	Corrected item-to-total correlation	Cronbach’s α if item deleted	Cronbach’s α
US	US1	0.639	0.750	0.806
	US2	0.666	0.721	
	US3	0.655	0.733	
PS	PS1	0.644	0.740	0.805
	PS2	0.658	0.726	
	PS3	0.651	0.733	
ER	ER1	0.591	0.730	0.779
	ER2	0.629	0.688	
	ER3	0.629	0.688	
UDH	UDH1	0.626	0.730	0.794
	UDH2	0.632	0.724	
	UDH3	0.650	0.705	
EE	EE1	0.632	0.731	0.796
	EE2	0.634	0.729	
	EE3	0.653	0.708	
TS	TS1	0.645	0.680	0.783
	TS2	0.604	0.725	
	TS3	0.616	0.713	
SC	SC1	0.662	0.721	0.804
	SC2	0.653	0.730	
	SC3	0.637	0.746	
ASS	ASS1	0.651	0.733	0.804
	ASS2	0.669	0.713	
	ASS3	0.633	0.751	
EF	EF1	0.638	0.766	0.812
	EF2	0.636	0.766	
	EF3	0.711	0.688	
SF	SF1	0.662	0.711	0.801
	SF2	0.621	0.753	
	SF3	0.655	0.718	
CUI	CUI1	0.646	0.747	0.808
	CUI2	0.650	0.744	
	CUI3	0.673	0.720	

Harman’s single-factor test is a widely used method for assessing common method bias (CMB). If CMB is present, all variables will load onto a single factor in an unrotated factor analysis, or a single factor will account for a substantial proportion of the variance^108^. Podsakoff and Organ suggest that if a single factor accounts for less than 50% of the variance in an unrotated exploratory factor analysis (EFA), CMB is likely within acceptable limits^109^. To examine for common method bias in this study, we employed Harman’s single-factor test using SPSS. As shown in Table 13, the results of the EFA revealed five factors with eigenvalues greater than 1, cumulatively explaining 59.175% of the variance. The first unrotated factor accounted for 44.321% of the variance, which is below the 50% threshold. This indicates that no single factor explains the majority of the variance, suggesting that common method bias is well-controlled in this study.

**Table 13:** Common variance explanation of driving assistance system (n = 504).

Element	Initial eigenvalue			Extract the sum of squares and load		
	Aggregate	Variable %	Cumulative %	Aggregate	Variable %	Cumulative %
1	14.626	44.321	44.321	14.626	44.321	44.321
2	1.489	4.514	48.834	1.489	4.514	48.834
3	1.198	3.631	52.465	1.198	3.631	52.465
4	1.176	3.565	56.029	1.176	3.565	56.029
5	1.038	3.145	59.175	1.038	3.145	59.175
6	0.990	3.000	62.174			
7	0.966	2.927	65.101			
8	0.858	2.601	67.702			
9	0.648	1.963	69.665			
10	0.595	1.804	71.469			
11	0.586	1.776	73.245			
12	0.558	1.690	74.935			
13	0.543	1.647	76.581			
14	0.517	1.567	78.149			
15	0.495	1.500	79.649			
16	0.489	1.483	81.131			
17	0.463	1.403	82.535			
18	0.454	1.376	83.911			
19	0.434	1.315	85.226			
20	0.431	1.305	86.531			
21	0.412	1.247	87.778			
22	0.405	1.228	89.006			
23	0.391	1.185	90.191			
24	0.388	1.176	91.366			
25	0.370	1.121	92.488			
26	0.361	1.094	93.582			
27	0.347	1.051	94.633			
28	0.341	1.033	95.666			
29	0.314	0.951	96.617			
30	0.301	0.911	97.528			
31	0.291	0.883	98.411			
32	0.271	0.823	99.234			
33	0.253	0.766	100.000			

Exploratory factor analysis was conducted using SPSS 22.0, employing the Kaiser–Meyer–Olkin (KMO) measure and Bartlett’s test of sphericity to ascertain the adequacy of correlations for factor extraction^110^. As shown in Table 14, the KMO values for the variables ranged from 0.701 to 0.721, all exceeding the threshold of 0.5, while the significance levels of Bartlett’s test were all below 0.05 and approached zero, indicating that the Bartlett’s test of sphericity was significant for all variables. This suggests a solid foundation for factor analysis of the data^111,112^. Consequently, principal component analysis was further utilized for factor analysis of the variables. The results indicated that each variable could extract only one factor with an eigenvalue greater than 1, and the cumulative variance contribution rates for all variables exceeded 50%. This demonstrates that the factors derived from the two scales in this study can adequately explain the variables, while the commonalities for all items were above 0.5, and the factor loadings were greater than 0.6, all within the recommended ranges proposed by previous research^113^. Therefore, this study concludes that the survey results exhibit strong unidimensionality.

**Table 14:** Results of exploratory factor analysis for driverless system (*n* = 504).

Dimension	Items	KMO	Bartlett sphere test	Factor Loading	Commonality	Eigenvalue	Total variation explained (%)
US	US1	0.721	0	0.855	0.731	2.223	74.103
	US2			0.866	0.750		
	US3			0.861	0.752		
PS	PS1	0.710	0	0.838	0.703	2.132	71.079
	PS2			0.844	0.713		
	PS3			0.847	0.717		
ER	ER1	0.701	0	0.817	0.667	2.079	69.305
	ER2			0.838	0.702		
	ER3			0.843	0.710		
UDH	UDH1	0.714	0	0.845	0.715	2.166	72.188
	UDH2			0.851	0.724		
	UDH3			0.853	0.727		
EE	EE1	0.707	0	0.832	0.693	2.113	70.448
	EE2			0.839	0.703		
	EE3			0.847	0.717		
TS	TS1	0.705	0	0.843	0.710	2.097	69.897
	TS2			0.832	0.693		
	TS3			0.833	0.694		
SC	SC1	0.712	0	0.844	0.713	2.145	71.512
	SC2			0.850	0.722		
	SC3			0.843	0.711		
ASS	ASS1	0.713	0	0.846	0.716	2.155	71.841
	ASS2			0.847	0.718		
	ASS3			0.849	0.721		
EF	EF1	0.716	0	0.845	0.714	2.216	73.862
	EF2			0.857	0.735		
	EF3			0.876	0.767		
SF	SF1	0.712	0	0.854	0.729	2.157	71.891
	SF2			0.836	0.698		
	SF3			0.854	0.729		
CUI	CUI1	0.716	0	0.849	0.722	2.184	72.808
	CUI2			0.848	0.719		
	CUI3			0.863	0.744		

Using AMOS for confirmatory factor analysis, the factor loadings of each item in the scale were found to be greater than 0.5, indicating that all items consistently explain their respective variables. This consistency not only suggests that the items effectively reflect the essence of the variables but also demonstrates that the scale possesses high stability and reliability in measuring these variables^114^. Consequently, calculations based on the factor loadings of each item yielded the composite reliability (CR) and average variance extracted (AVE) values. The CR should not fall below 0.7, while the minimum standard for AVE is 0.5^115,116^. As shown in Table 15, the factor loadings in this study are all greater than 0.5, with CR values exceeding 0.78, and the square roots of AVE values are all greater than 0.541, indicating that the relevant variables exhibit good convergent validity.

**Table 15:** Convergent factor analysis results of driverless system (*n* = 504).

Dimension	Items	Unstandardized factor loading	Standardize factor loading	SE	*P*-value	AVE	CR
US	US1	1	0.769	–	–	0.580	0.805
	US2	0.946	0.744	0.052	0		
	US3	0.995	0.771	0.053	0		
PS	PS1	1	0.766	–	–	0.579	0.805
	PS2	0.983	0.762	0.053	0		
	PS3	0.967	0.754	0.052	0		
ER	ER1	1	0.715	–	–	0.541	0.780
	ER2	1.066	0.768	0.062	0		
	ER3	1.010	0.723	0.062	0		
UDH	UDH1	1	0.760	–	–	0.562	0.794
	UDH2	0.931	0.724	0.054	0		
	UDH3	0.995	0.764	0.054	0		
EE	EE1	1	0.754	–	–	0.566	0.796
	EE2	1.002	0.757	0.055	0		
	EE3	1.003	0.746	0.056	0		
TS	TS1	1	0.753	–	–	0.547	0.784
	TS2	0.927	0.724	0.053	0		
	TS3	0.978	0.741	0.055	0		
SC	SC1	1	0.758	–	–	0.578	0.804
	SC2	1.054	0.761	0.057	0		
	SC3	1.034	0.762	0.055	0		
ASS	ASS1	1	0.753	–	–	0.579	0.805
	ASS2	1.052	0.796	0.055	0		
	ASS3	0.940	0.732	0.054	0		
EF	EF1	1	0.754	–	–	0.594	0.814
	EF2	0.873	0.722	0.052	0		
	EF3	1.060	0.832	0.053	0		
SF	SF1	1	0.760	–	–	0.573	0.801
	SF2	0.924	0.746	0.052	0		
	SF3	0.994	0.765	0.054	0		
CUI	CUI1	1	0.756	–	–	0.584	0.808
	CUI2	1.043	0.777	0.056	0		
	CUI3	0.980	0.759	0.054	0		

0.5 is the minimum standard for AVE, and CR ≥ 0.7

Discriminant validity refers to the distinction between different latent variables. According to Fornell and Larcker’s recommendations, all values should not exceed 0.9, indicating that the scale possesses good discriminant validity^116^. As shown in Table 16, the latent variables exceed the correlation coefficients among the latent variables, suggesting that there is significant correlational information among the main variables, and none exceed 0.9. Furthermore, the correlations of the exogenous structures are all less than 0.85^117^, indicating that both testing models exhibit good discriminant validity.

**Table 16:** Correlation coefficient and mean extraction variance of driverless system (*n* = 504).

Latent variable	ASS	CUI	EE	EF	ER	PS	SC	SF	TS	UDH	US
ASS	0.848										
CUI	0.805	0.850									
EE	0.811	0.796	0.843								
EF	0.791	0.783	0.807	0.852							
ER	− 0.793	− 0.775	− 0.787	− 0.773	0.833						
PS	0.818	0.797	0.812	0.801	− 0.797	0.848					
SC	0.833	0.789	0.821	0.814	− 0.812	0.821	0.848				
SF	0.803	0.782	0.802	0.784	− 0.795	0.792	0.792	0.846			
TS	0.794	0.796	0.797	0.797	− 0.807	0.810	0.809	0.805	0.835		
UDH	0.803	0.806	0.806	0.771	− 0.777	0.810	0.810	0.780	0.800	0.841	
US	0.803	0.805	0.792	0.803	− 0.790	0.819	0.825	0.778	0.798	0.795	0.849

### Model testing

Relevant research indicates that the SRMR value is one of the indices for assessing model fit; a smaller SRMR value signifies a better fit of the model to the data. When the SRMR value is below 0.08, it indicates a satisfactory fit of the model to the data^118,119^. An d-ULS value less than 0.95 suggests a good fit of the model to the data, with a moderate number of free parameters^120^. Similarly, a d-G value below 0.95 also indicates a good fit of the model to the data, along with a reasonable number of free parameters^121^. Furthermore, an NFI greater than 0.8 signifies a high model fit, with values closer to 1 indicating a stronger fit, demonstrating the model’s ability to accurately capture the structures and relationships within the data^122^. Therefore, as shown in Table 17, the SRMR value of 0.041 is below the threshold of 0.08, the d-ULS value of 0.925 and the d-G value of 0.749 are both below the threshold of 0.95, and the NFI value of 0.836 exceeds 0.8, indicating a good fit for the model of the driverless system.

**Table 17:** Model fitting measures for driverless system.

Common indices	d-ULS	d-G	SRMR	NFI
Criteria	< 0.95	< 0.95	< 0.08	> 0.8
Values	0.925	0.749	0.041	0.836

### Path hypothesis analysis

Based on the results from Table 15 regarding the path coefficient tests of the user’s continuous usage intention for driverless systems, it is evident that user scale has a positive and significant impact on perceived importance (T = 59.349, *P* < 0.05), experience regret (T = 47.916, *P* < 0.05), and user driving habits (T = 6.612, *P* < 0.05), thereby supporting hypotheses H10/H11/H12. Furthermore, perceived importance positively and significantly influences user driving habits (T = 8.757, *P* < 0.05) and continuous usage intention (T = 1.981, *P* < 0.05), providing support for hypotheses H13/H15. Experience regret has a negative significant impact on user driving habits (T = 6.041, P < 0.05), supporting hypothesis H14. However, it shows a non-significant negative correlation with continuous usage intention (T = 1.525, *P* > 0.05), indicating that hypothesis H16 is not supported. User driving habits positively and significantly influence continuous usage intention (T = 4.183, *P* < 0.05), thus supporting hypothesis H17. The research findings also indicate that technical stability (T = 2.623, *P* < 0.05), after-sales service (T = 3.469, *P* < 0.05), and ethical factors (T = 2.432, *P* < 0.05) directly affect users’ continuous usage intention. In contrast, economic benefits (T = 1.863, *P* > 0.05), safety capabilities (T = 0.346, *P* > 0.05), and social factors (T = 1.931, *P* > 0.05) do not have a direct impact. As summarized in Table 18, the results of the hypothesis tests are presented.

**Table 18:** Path relationship testing of hypothetical models for driverless system.

Hypothesis	Relationship	Coefficient	T statistics	*P* values	Results
H2	US—> PS	0.819	59.349	0	Accept
H4	US—> ER	− 0.790	47.916	0	Accept
H6	US—> UDH	0.294	6.612	0	Accept
H8	PS—> UDH	0.370	8.757	0	Accept
H10	PS—> CUI	0.099	1.981	0.048	Accept
H12	ER—> UDH	− 0.250	6.041	0	Accept
H14	ER—> CUI	− 0.067	1.525	0.127	Not accept
H16	UDH—> CUI	0.198	4.183	0	Accept
H18a	EE—> CUI	0.102	1.863	0.063	Not accept
H18b	TS—> CUI	0.122	2.623	0.009	Accept
H18c	SC—> CUI	0.018	0.346	0.730	Not accept
H18d	ASS—> CUI	0.160	3.469	0.001	Accept
H18e	EF—> CUI	0.111	2.432	0.015	Accept
H18f	SF—> CUI	0.087	1.931	0.054	Not accept

**Fig. 5:**
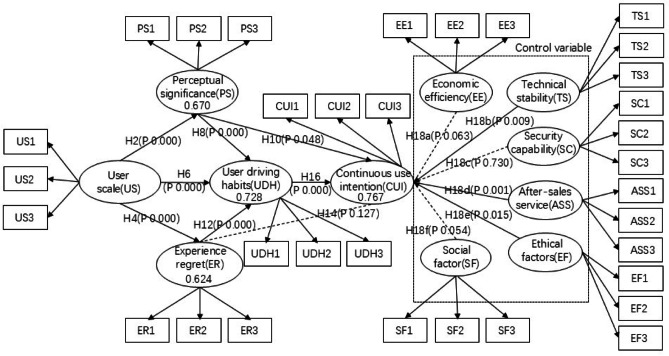
Hypothesis test results for driverless system.

According to the significant hypothesis testing results presented in the table, as shown in Fig. 5

## Discussion

This study is based on the extended Technology Acceptance Model and incorporates control variables to conduct a comparative analysis of users’ continuous intention to use two distinct types of autonomous driving systems: assisted driving systems and driverless systems. The research utilizes the same structural equation model and questionnaire items to analyze data from users who have experienced assisted driving systems as well as those who have used or experienced driverless systems. Additionally, the statistical results from Study 1 and Study 2 are compiled for comparative discussion, leading to the following conclusions:

With the continuous advancement of automotive autonomous driving systems, users’ experiences and interactions with different driving systems have sparked significant divergence in the market regarding the developmental direction of automotive driving systems. Some scholars advocate that automotive driving systems should evolve towards assisting users in driving, as they place greater trust in the reliability of human driving skills^123,124^. Conversely, another group of scholars argues that automotive driving systems should progress towards driverless systems, thereby initiating a new phase of technological innovation^125^. The discussions surrounding these differing viewpoints fundamentally revolve around the actual utility value for users. Therefore, it is essential to investigate the relationship between different driving systems and users’ intentions for continued use, while also incorporating relevant control variables to explore the factors influencing users’ sustained usage intentions. This not only provides guidance for the development of various driving systems but also offers insights for future explorations of these two distinct types of driving systems. This study constructs two models of users’ continued usage intentions for different driving systems based on habit theory and regret theory, aiming to examine the impact mechanisms of user scale, perceived importance, and experience-related regret on users’ driving habits and sustained usage intentions. By researching the same variables across different driving systems, we gain a deeper understanding of users’ intentions for continued use of these two systems. The research employs an online survey method, targeting users of assisted driving systems and users in cities with driverless taxi services, and utilizes structural equation modeling to analyze the sample data.

1. Based on the hypothetical data, among the variables of the advanced driver-assistance systems, user perceived importance (T = 4.283) and user driving habits (T = 4.283) significantly influence users’ intentions for continued use, indicating that perceived importance and user habits are critical factors affecting users’ ongoing usage intentions. According to the perspective that a higher T value indicates greater significance^126^, although perceived importance (T = 8.757), regret after experience (T = 6.041), and user scale (T = 6.612) all have significant impacts on user driving habits, the influence of perceived importance on user driving habits is particularly pronounced. In the data concerning driverless systems, the variable that most significantly affects users’ intentions for continued use is user driving habits (T = 4.183), suggesting that user driving habits are a crucial determinant of continued use of driverless systems. Furthermore, there is a significant correlation between the relevant variables and user driving habits, with perceived importance (T = 4.183) having the most substantial impact on user driving habits. A comparison of the data from advanced driver-assistance systems and driverless systems reveals that users perceive perceived importance and driving habits as equally important in the context of advanced driver-assistance systems; however, in driverless systems, perceived importance becomes more critical. This is attributed to the fact that users do not need to control the vehicle when using driverless, whereas advanced driving assistance requires actual user operation. Therefore, the results of the data hypothesis are reasonable. Additionally, the significance of perceived importance on user driving habits among the relevant variables is notably high, aligning with Silvia’s assertion regarding the significant role of perceived importance in driving habits^127^.
2. Both data models indicate that user-perceived importance and experience regret significantly influence user driving habits. From the perspective of data significance, the impact of user-perceived importance on driving habits is the most pronounced in both models, and it also significantly affects users’ intentions to continue using the system. However, while experience regret has a significant effect on driving habits, it does not hold true for the hypothesis regarding continued usage intention. This suggests that users’ intentions to continue using the system, based on rational perceptions, are clear and affirmative, whereas emotional experiences do not play a similar role. This viewpoint aligns with Davis’s assertion that user perception has a significant correlation with both current and future usage^44^. It indicates that users of both types of driving systems are more focused on practical usage functions. Therefore, whether it is an advanced driver-assistance system or a driverless system, user promotion and system improvements should center around the actual user experience, taking into account the practical aspects of different types of driving systems. Although there is no significant direct relationship between experience regret and continued usage intention, it is valid that experience regret indirectly influences continued usage intention through user driving habits, which also reflects the rationale behind the establishment of the experience regret variable. Furthermore, the mechanism by which user scale affects continued usage intention operates through perceived importance, experience regret, and user driving habits, which have direct or indirect impacts on users’ intentions to continue using the system. This study seizes the opportunity to explore and validate the theoretical mechanisms of influence between user scale and continued usage intention in the context of different types of autonomous driving systems.
3. The significance of control variables varies considerably between the two models. In the model for the advanced driver-assistance systems (ADAS), economic benefits (T = 2.027, *P* < 0.05), technological stability (T = 3.089, *P* < 0.05), ethical considerations (T = 4.433, *P* < 0.05), and social impact (T = 2.233, *P* < 0.05) significantly influence users’ intentions to continue using the ADAS. Conversely, the safety of the system and after-sales service do not significantly affect users’ intentions to persist in using the system. This indicates that users’ decisions to continue utilizing the ADAS are not swayed by the system’s inherent safety or the quality of after-sales service, suggesting that users place greater trust in their own control over the vehicle and show less concern for the system’s maintenance. This perspective aligns with Sina’s assertion regarding the substantial differences between automated driving systems and driver behavior^128^. In the model for driverless systems, technological stability (T = 2.623, *P* < 0.05), after-sales service (T = 3.469, *P* < 0.05), and ethical factors (T = 2.432, *P* < 0.05) significantly impact users’ intentions to continue using the system, while economic factors, safety, and social influence do not have a significant effect. This suggests that users do not consider the costs and energy efficiency associated with using driverless systems, likely due to the inherent energy-saving and environmentally friendly characteristics of these systems, which are designed for optimal routing and driving efficiency compared to manual driving. Additionally, the lack of significant impact of system safety on users’ intentions to continue using the system parallels Zhang’s view that safety does not significantly influence drivers during automated driving ^129^, indicating that the safety of the system itself is not a primary concern for users. Moreover, the impact of social influence on users’ intentions for continued use is not significant, which may be attributed to the fact that the use of driverless systems is a personal behavior, less affected by the surrounding environment and others. This perspective aligns with Anol’s assertion that while social influence and the perceived safety of driverless technology have a positive correlation with usage intention, during the continued use phase, other factors such as technology acceptance and personal preferences become more critical, supporting the notion that personal behavior is not influenced by the surrounding environment and others^82^.

**Fig. 6:**
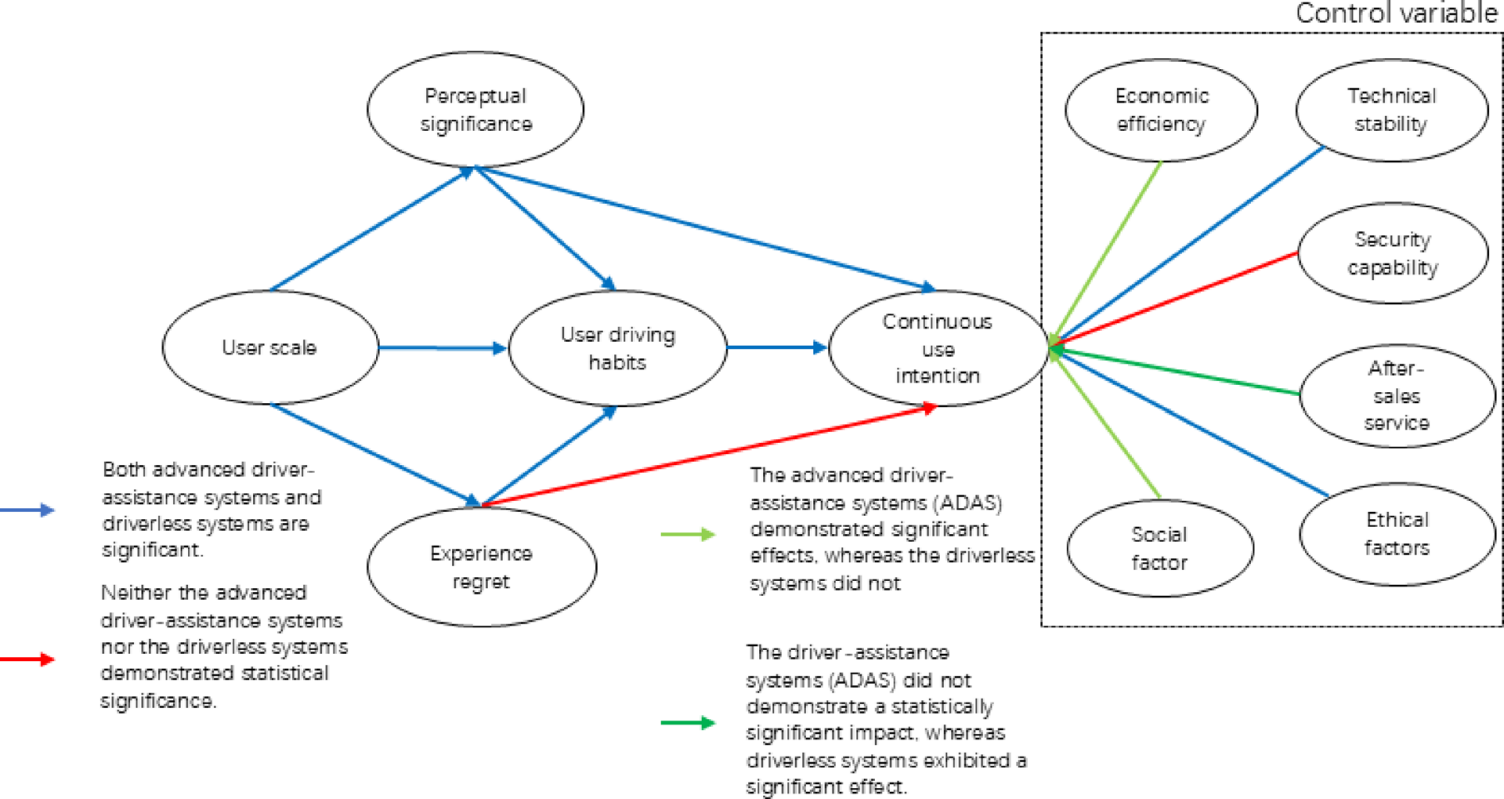
Comparative analysis of the significance of Advanced Driver-Assistance Systems (ADAS) versus driverless systems.

As illustrated in Fig. 6, the comparison between the two models is made more intuitive through the use of differently colored arrows. The most significant divergence is observed in the control variables. Specifically, users of advanced driver-assistance systems (ADAS) exhibit a greater emphasis on economic factors and social impacts, while placing less importance on after-sales service. Conversely, users of driverless driving systems demonstrate a heightened concern for after-sales service, with comparatively less focus on economic factors and social impacts. The conclusions drawn from the two system models indicate that both types of driving systems recognize the importance of perceived significance in influencing users’ driving habits and their intentions for continued use. Regarding the factors affecting users’ intentions for sustained usage, ADAS users are more concerned with economic benefits, technological stability, ethical considerations, and social impacts during the system’s operation. Conversely, users of driverless systems prioritize technological stability, after-sales service, and ethical factors. While both systems share common characteristics, they also exhibit distinct differences. In the development of these systems, it is essential to enhance and refine each system based on the specific concerns of their respective users, thereby enabling each system to provide more efficient services and improve the social traffic environment.

## Theoretical and implications

This study focuses on the factors influencing users’ continuous usage intentions of different types of driving systems and the underlying mechanisms, establishing a comprehensive model framework. This framework encompasses core variables such as user scale, perceived importance, experience regret, and user driving habits, while also incorporating control variables related to economics, technology, safety, after-sales service, ethics, and social aspects. The findings indicate that user scale, perceived importance, and experience regret significantly impact user driving habits, with perceived importance and experience regret not only directly influencing users’ continuous usage intentions but also exerting indirect effects. User driving habits serve as a critical mediating variable, acting as a vital pathway through which user experience, perceived importance, and experience regret continue to exert their influence.

1. From the perspective of theoretical contribution, this research holds significant importance. It positions perceived importance, experience regret, and user scale as antecedent variables, with user driving habits as a mediating variable, while integrating control variables related to economics, technology, safety, after-sales service, ethics, and social factors to construct a novel theoretical framework. This innovation not only opens new avenues for behavioral research in habit theory and experience regret theory but also validates a theoretical model of users’ continuous usage intentions applicable to various types of autonomous driving systems in China, further enriching and refining the theoretical system in the relevant field.
2. In terms of practical significance, the findings of this study provide valuable insights and references for the improvement and development of various types of automotive autonomous driving system technologies. Enhancing users’ intention for continued use is a core direction for future advancements in automotive driving systems. It is essential to closely monitor the development trends of relevant control factors and to make precise improvements and optimizations based on the market environment and user needs of different driving systems. Furthermore, during the promotion of various systems, it is crucial to emphasize the user experience of perceived usefulness and perceived ease of use, which will help users develop good driving habits and, in turn, strengthen their intention for continued use. Additionally, specific improvement measures should fully consider the personalized needs of different user groups under various systems to achieve targeted optimization.
3. From a future development perspective, this study centers on user experience, aiming to comprehensively enhance the service quality of different driving systems and create a superior driving experience for users. In subsequent research, it would be beneficial to further explore the impact and mechanisms of different control variables, as well as conduct detailed scientific segmentation studies of user groups based on gender and age. This approach will enable a more accurate understanding of user needs and facilitate the continuous upgrading and refinement of driving systems.

In essence, this study elucidates the driving factors behind users’ sustained utilization of diverse driving systems, thereby offering a theoretical foundation and practical implications for the optimization and promotion of these systems. The theoretical model developed in this research exhibits commendable generalizability and stability, providing a valuable framework for subsequent investigations. The findings are not constrained by the specific research subjects, offering a universally applicable theoretical model for the study of user continuance intention in various autonomous driving systems, thus contributing to both the academic discourse and practical advancements within the autonomous driving domain.

## Conclusions

This study focuses on Advanced Driver-Assistance Systems (ADAS) and driverless systems, conducting an in-depth comparative analysis of users’ continuous usage intentions. The aim is to identify key factors influencing user behavior, with the research findings offering valuable insights and references for the development of autonomous driving systems. The research reveals both commonalities and significant differences in the mechanisms influencing users’ continuous usage intentions for these two systems. A comparative analysis of user intentions for ADAS and driverless systems reveals both similarities and differences. Regarding similarities, perceived importance and regret of experience significantly impact users’ driving habits, with perceived importance notably influencing continuous usage intentions. This indicates that users prioritize rational functional value. Although regret of experience does not directly affect continuous usage intentions, it indirectly influences them through driving habits. User scale indirectly affects continuous usage intentions through perceived importance, regret of experience, and driving habits. Regarding differences, in ADAS, perceived importance and driving habits equally influence continuous usage intentions. Among the control variables, economic benefits, technical stability, ethical factors, and social impact are significant, while safety and after-sales service are not. Users are more concerned with economic and social impacts. In driverless systems, driving habits are the key factor influencing continuous usage intentions, with perceived importance having the most prominent impact on driving habits. Among the control variables, technical stability, after-sales service, and ethical factors are significant, while economic factors, safety, and social impact are not. Users are more concerned with after-sales service. Overall, both systems need to emphasize perceived importance, technical stability, and ethical factors. Additionally, ADAS should focus on improving economic benefits and social acceptance, while driverless systems should prioritize enhancing after-sales service to optimize user experience and promote improvements in the traffic environment.

Furthermore, this study has certain limitations. During the sample collection process, issues of age bias and regional bias emerged. In terms of age bias, the online questionnaire method may have resulted in a higher proportion of young and middle-aged respondents, while the representation of the elderly population was relatively low. This imbalance fails to adequately reflect the attitudes and acceptance levels of the elderly towards Driverless systems. Regarding regional bias, the questionnaire collection was primarily concentrated in first-tier cities such as Beijing, Shanghai, and Guangzhou, making it difficult to represent the perspectives and needs of users from other regions. Given the trend of an aging society in China, the awareness and acceptance of autonomous driving systems among the elderly require urgent attention. Older adults typically exhibit a slower acceptance rate of emerging technologies, necessitating targeted research and promotional efforts.

For future research improvements, it is essential to expand the sample range and employ diverse data collection methods, such as offline surveys and interviews, to obtain a more representative sample that encompasses users of varying ages, regions, and backgrounds. Additionally, in-depth studies focusing on the elderly population should be conducted to understand their acceptance willingness, concerns, and expectations regarding autonomous driving systems, as well as to explore effective promotional strategies. Longitudinal studies could also be undertaken to track users’ experiences and attitude changes towards autonomous driving systems, thereby providing deeper insights into the dynamic process of sustained user behavior. Consequently, while this research offers valuable insights into understanding users’ intentions for continued use of autonomous driving systems, its limitations must not be overlooked. Future studies should address these limitations to achieve a more comprehensive and profound understanding, ultimately guiding the development and promotion of autonomous driving systems more effectively.

## Data Availability

The data are contained within the article.
